# Web-based NGS data analysis using miRMaster: a large-scale meta-analysis of human miRNAs

**DOI:** 10.1093/nar/gkx595

**Published:** 2017-07-12

**Authors:** Tobias Fehlmann, Christina Backes, Mustafa Kahraman, Jan Haas, Nicole Ludwig, Andreas E. Posch, Maximilian L. Würstle, Matthias Hübenthal, Andre Franke, Benjamin Meder, Eckart Meese, Andreas Keller

**Affiliations:** 1 Chair for Clinical Bioinformatics, Saarland University, Saarbrücken, Germany; 2 Hummingbird Diagnostics GmbH, Heidelberg, Germany; 3 Department of Internal Medicine III, University Hospital Heidelberg, Heidelberg, Germany; 4 German Center for Cardiovascular Research (DZHK), Heidelberg, Germany; 5 Klaus Tschira Institute for Integrative Computational Cardiology, Heidelberg, Germany; 6 Department of Human Genetics, Saarland University, Homburg, Germany; 7 Ares Genetics GmbH, Vienna, Austria; 8 Siemens Healthcare GmbH, Strategy and Innovation, Erlangen, Germany; 9 Institute of Clinical Molecular Biology, Christian-Albrechts-University of Kiel, Kiel, Germany

## Abstract

The analysis of small RNA NGS data together with the discovery of new small RNAs is among the foremost challenges in life science. For the analysis of raw high-throughput sequencing data we implemented the fast, accurate and comprehensive web-based tool miRMaster. Our toolbox provides a wide range of modules for quantification of miRNAs and other non-coding RNAs, discovering new miRNAs, isomiRs, mutations, exogenous RNAs and motifs. Use-cases comprising hundreds of samples are processed in less than 5 h with an accuracy of 99.4%. An integrative analysis of small RNAs from 1836 data sets (20 billion reads) indicated that context-specific miRNAs (e.g. miRNAs present only in one or few different tissues / cell types) still remain to be discovered while broadly expressed miRNAs appear to be largely known. In total, our analysis of known and novel miRNAs indicated nearly 22 000 candidates of precursors with one or two mature forms. Based on these, we designed a custom microarray comprising 11 872 potential mature miRNAs to assess the quality of our prediction. MiRMaster is a convenient-to-use tool for the comprehensive and fast analysis of miRNA NGS data. In addition, our predicted miRNA candidates provided as custom array will allow researchers to perform in depth validation of candidates interesting to them.

## INTRODUCTION

MicroRNAs (miRNAs) play a central role in orchestrating human gene regulation and are consequently prime targets in biomedical research. Many miRNAs from *Homo sapiens* and other species are collected in the miRBase (1). Currently, the fraction of actually true positive miRNAs in this database is controversially discussed (2–10), especially later versions seem to contain many false positives (11). On the one hand, this calls for curated databases, on the other hand not all miRNAs, especially context specific ones, seem to be discovered yet.

Various experimental approaches are applied for measuring miRNA expression levels including approaches for small sets of selected miRNAs like RT-qPCR, CMOS based assays (12) or immunoassays (13). The most frequently employed genome-wide assays include microarray screening and high-throughput sequencing (HT-seq). A comparison of 12 different experimental approaches is provided by Mestdagh *et al*. (14).

HT-seq enables—beyond quantitative analysis of known miRNAs—single-base resolution of known and novel miRNAs (15) and thus is currently applied to discover the afore mentioned context-specific miRNAs. For the analysis of HT-seq data, a wide range of stand-alone and web-based bioinformatics tools have been implemented allowing the prediction of novel miRNA candidates and quantification of miRNAs (16,17), detection of miRNA isoforms (18,19), miRNA set enrichment analyses (20,21), and prediction of miRNA targets (22,23) among others. Akthar *et al*. published a comprehensive review on 129 available miRNA bioinformatics tools (24). The different data formats used in these tools and the challenges to combine web-based and stand-alone solutions, however, complicate the design of integrated pipelines.

Our ambition was to develop a web-based application that combines the most frequently requested analyses. An important aspect of our tool termed miRMaster (www.ccb.uni-saarland.de/mirmaster) was to facilitate HT-seq data analysis of human samples from raw sequencing files provided in the FASTQ format. Building up on the basic principle of miRDeep2 (16) as the most frequently used prediction tool for miRNAs, we implemented an own predictor with an extended feature set including our previously developed prediction score (11). Furthermore, we implemented functionality to report the presence of miRNA motifs to the user (25–27). MiRMaster allows to search for novel miRNA candidates, to quantify miRNA expression, to identify isoforms and variants of miRNAs. Another feature of miRMaster is the mapping of non-human small RNA reads against the NCBI RefSeq collection of bacterial and viral genomes (28), thereby allowing the detection of contaminations, infections or exogenous miRNAs. To allow the analysis of targets regulated by miRNAs, we implemented Application Programming Interfaces (APIs) to available web-based tools for considering the targetome (miRTargetLink (29)) and to carry out miRNA set enrichment (miEAA (20)).

Since different research groups measured various specimens using different experimental protocols and bioinformatics pipelines and not all data stored in a central repository, a redundancy between the studies exist. Besides the miRNAs in the miRBase, and specific studies mentioned before, several comprehensive analyses (e.g. Londin *et al*. (30), Backes *et al*. (11), Friedländer *et al*. (31), Jha *et al*. (32)) propose hundreds to thousands of new miRNAs. To detect as many as possible miRNA candidates we performed a comprehensive analysis of 1836 data sets containing 20 billion reads.

## MATERIALS AND METHODS

### Sample collection

As case study we analyzed an in-house NGS miRNA sample collection of 1097 samples from blood and blood cell components (33–39). Further we downloaded 739 samples from four series of the GEO database (40): GSE64142, GSE53080, GSE49279 and GSE45159. All samples have been sequenced using Illumina Next-Generation sequencing. Table 2 presents an overview of these samples including a description, number of samples, number of reads and file size.

### Positive miRNA dataset for training miRMaster

A straightforward positive dataset would consist of the complete miRBase (1). However, others and we have observed that miRBase may contain false positives, especially in the last versions (41). Therefore, we selected all miRNA precursors from miRBase 1 to 7 and all precursors of miRNAs containing strong experimental evidence in the miRTarBase (42), leading to 487 high-confidence positive miRNAs. We defined precursors by their 5′ and 3′ mature miRNAs, i.e. they start with the first base of the 5′ miRNA and end with the last base of the 3′ miRNA. For miRBase precursors that had only one form annotated we derived the other from its hairpin, as described for our prediction algorithm. Therefore, our predictions are independent of the size of the stem loops provided in miRBase.

### Negative miRNA dataset for training miRMaster

Choosing an appropriate negative dataset is a challenging task, since miRNAs can be located anywhere in the genome (43). A correct negative dataset plays an important role for the creation of a well-trained classifier. Overall, since only a small fraction of the genome and of sequences that form hairpins are actually precursors, we built five different sets to cover as many potential wrong predictions as possible. The different negative datasets were derived from separate assumptions and combined for our training procedure. The first dataset was built to cover predictions, where one actual miRNA is contained in the predicted precursor but the other miRNA is wrongly annotated. We assume that real precursors do not overlap. It was created by splitting in half all known stem–loops from miRBase that contained two annotated mature miRNAs. We adjusted the length to the original stem-loop by including the flanking regions. To determine the positions of the miRNAs in the two new pseudo precursors, we kept the original miRNAs and derived the other based on it, as in our prediction algorithm. This dataset was composed of 298 precursors. The second dataset was created to cover predictions that could stem from protein coding sequences of genes without known alternative splicing events. It was derived from the widely used pseudo precursor set built by Xue *et al.* (44). We first kept only sequences that aligned perfectly to the latest assembly of the human genome (hg38). Then we segmented these sequences to enable the computation of segment specific features. Therefore, we determined the position of one of the pseudo miRNAs by assigning it to the segment with most base pairs, having a length of 20 nucleotides and non-overlapping with the loop region. The other was derived from it, as in our predicting algorithm. The resulting set contained 3916 pseudo precursors. The third dataset was created to cover predictions that could arise from stem-loops of other ncRNAs. It was shown by others (45) that for a very small portion of all known miRNAs this could actually be the case. However, due to their low number and the false positives largely outweighing the true positives we considered this set to be useful to reduce the false positive prediction rate. The dataset was derived from Rfam (46) (release 11) and composed of 3342 negative precursors. We considered all human ncRNAs that were not miRNAs and derived pseudo precursors by retaining only those that could be partitioned into 5′, 3′ and loop parts. The fourth dataset was created to account specifically for predictions that would pass the filtering steps in our algorithm, but which would overlap with other ncRNAs. It is in fact an extension of the third dataset. We derived 4031 pseudo precursors by running our prediction on 705 in-house samples and keeping only those that passed all filtering steps but overlapped with other ncRNAs of Rfam. The fifth dataset was created to account for predictions that were not covered by the other negative datasets. It was derived from early predictions performed by our algorithm (trained on the other four datasets) on our in-house samples. This set addresses specifically predictions where the miRNAs contained many repeated bases and further, miRNA duplexes with high normalized free energy and precursors with high normalized free energy. We kept all predictions that displayed evidence for being false positives, i.e. precursors with miRNAs containing at least seven consecutive A or U or 8 C or G. Further we kept all with a normalized ensemble free energy of over –0.15 kcal/mol*nt or with a normalized duplex minimum free energy of over –0.15 kcal/mol*nt. The cutoffs were determined empirically by analyzing the distribution of the properties of known precursors. This led to 797 additional negative miRNAs. For the first four datasets we further retained only those pseudo precursors without bifurcations, with at least 50% paired bases between the 5′ and 3′ pseudo miRNAs and with a 5′-3′ miRNA length difference of at most 10. The combination of all negative datasets resulted in 12 384 pseudo precursors, which are listed in Supplementary Table S2.

### Independent test sets for evaluating miRMaster

To validate the performance of our model we created two additional independent test sets. The first set was composed of human precursors of MirGeneDB (10) that were not used in our training process, resulting in 129 precursors. For the pseudo precursors we selected all sequences that were annotated as human precursors in earlier miRBase versions (1–20) and that were not duplicates or merged with known precursors. This resulted in 28 sequences, of which 6 were discarded by our algorithm when trying to determine a valid corresponding second miRNA arm. In addition, we created a second set composed of mouse precursors of MirGeneDB that had different sequences than our training precursors, resulting in 350 precursors. We selected the negative set analogously to the first negative set from early annotated mouse precursors, leading to 65 sequences. We mapped those sequences against the mouse genome (mm10) and removed all sequences which were not found or found at multiple positions. Of the remaining 56 sequences, 11 were discarded by our algorithm when trying to determine a valid second miRNA.

### Features of miRMaster for predicting novel miRNAs

We created a feature set composed of 216 properties, based on 186 existing features described in (44,47–51) and 30 novel features. Novel features included our previously developed novoMiRank score (11), open/close parentheses and unpaired nucleotides in all thirds of a precursor, 5′-3′ miRNA duplex minimum free energy, the number of base pairs in the 5′ and 3′ miRNAs and in-between, and the nucleotide ratio of the 5′ and 3′ miRNAs. Supplementary Table S1 lists all features including a brief description, their runtime impact and the *P*-value resulting from a two sided Wilcoxon rank-sum test after Benjamini–Hochberg adjustment for multiple testing (52) (alpha = 0.05) on our positive and negative datasets.

### Classifier selection for predicting miRNAs

To obtain the best classifier for our positive and negative dataset in terms of specificity and sensitivity we evaluated 180 different combinations of feature scaling, subset selection and classification methods using the scikit-learn Python toolkit (53), as shown in Supplementary Table S9. Since a large fraction of features can be computed in minimal time while very few features take very much computing time we built two models: one is based on all features and one based on the features with low runtime. For each combination we tuned the classifier's hyper-parameter via particle swarm optimization towards maximum ROC AUC, resulting in a total of 130,105 models. From those we then selected all models that performed at least as good as the best 25% according to ROC AUC, Precision-Recall AUC, sensitivity, specificity and Matthews correlation coefficient (MCC). The final model was chosen according to the highest F_0.5_ measure. Supplementary Figure S15 sketches this process.

### Input data of users to miRMaster

Since our ambition was to facilitate comprehensive miRNA analysis for all researchers, we implemented upload functionality for FASTQ files that are processed and compressed in the browser before being sent to the server. Thus, no additional software installation that compresses the files on the user's computer is needed. This feature is supported by only few tools, such as MAGI (54). Further we provide support for gzip compressed FASTQ files, since they are the typical storage format of sequencing files, thereby obviating the need to decompress files before inputting them to miRMaster.

### Preprocessing

Before sending the input files to our server we perform three preprocessing steps consisting of adapter trimming, quality filtering and read collapsing. Adapter trimming is performed via fuzzy string matching and can be customized by the user. We allow one mismatch and require an overlap of at least 10 nucleotides with the read per default. Further the user has the possibility to trim leading and trailing *N*, discard reads containing any remaining *N* and remove reads shorter than a specific size. For the quality filtering step, we re-implemented the sliding window filtering approach used by Trimmomatic (55). This allows reducing the amount of data sent by up to 99.9% (depending on the sample specimens). To take advantage of multi-core processor capabilities we use JavaScript web workers to allow the preprocessing of multiple files at the same time.

### Mapping to various ncRNA databases

We map the collapsed reads using Bowtie (56) and allow per default no mismatches against human rRNAs, snRNAs, snoRNAs, scaRNAs and lincRNAs of the Ensembl non-coding RNA database (release 85) (57), against piRNAs of piRBase (1.0) (58) and tRNAs of GtRNAdb (59). This allows the user to easily verify if the distribution of reads is as expected or to investigate specific RNAs. To allow the user to investigate specific ncRNAs we provide detailed expression counts for all ncRNAs we are mapping against, as well. The expression is determined by the number of reads mapping to a specific sequence using Bowtie. Further we report the mapping of reads against the human miRBase (version 21), which can be used to estimate the potential of finding novel miRNAs in the samples.

### Mapping to reference

Mapping the collapsed reads to the reference genome is performed using Bowtie. Analogous to miRDeep2 (16), we require no mismatches in the first 18 nucleotides and discard reads that map to over five different locations.

### Precursor excision, segment determination and filtering

The precursor excision, segment determination and filtering according to their structure and signature is performed analogous to miRDeep2. Briefly, local maximum read stacks in downstream windows of 70 nucleotides are searched and two precursors excised from each stack. The secondary structure is computed for each precursor using RNAfold (60). The maximum read stack represents one miRNA of the precursor. The other miRNA is determined by the paired sequence on the other arm with a 2-nucleotide overhang. Filtering steps are composed of a structure and signature filter. The secondary structure is required to have no bifurcations, a minimum percentage of base pairs in the highest expressed miRNA of 60% and a length difference of both miRNAs of at most five nucleotides. The signature is checked by mapping all reads with at most one mismatch against all excised precursors. At least 90% of all reads need to map to either a miRNA or in between, thereby discarding reads that do not map according to Dicer processing. All these thresholds can be customized in the web interface.

### Feature computation and prediction

After the potential precursors have been excised and filtered we compute their feature values and perform the prediction using our classifier as described in previous parts of the Materials and Methods section.

### Prediction merging and global signature filtering

Once the predictions for all samples have been performed we merge the resulting potential precursors in order to avoid multiple predictions shifted by only a few bases. Therefore, we group all precursors that differ by at most 10 positions and keep the one that was found in most samples. To make use of additional information provided by multiple samples we first normalize the expression of each read of each sample to reads per million (RPM) and sum up identical reads. Then we map the normalized reads of all samples against the merged predictions and score their signature. We weight each read using the following formula

\begin{eqnarray*} &&score\left( {read} \right) \\ &&= \ total\_RPM\left( {read} \right) \cdot length\left( {read} \right) \cdot \sqrt {\frac{{occuring\_samples\left( {read} \right)}}{{\# total\_samples}}} \end{eqnarray*}

Thereby, we penalize reads that occur in only few samples while giving more weight to longer reads. Reads mapping with mismatches are penalized per default by a dividing factor if they occur in at most 10% of all samples (but at most 10 samples). The dividing factor is the limit of occurring samples minus 1, but at least 2. We then remove all predictions that have a signature with an inconsistent dicer processing read portion representing at most 20% of the total score.

### Categories of new miRNAs

We assign to each predicted precursor one of six categories. (1) *Known*: when the prediction is overlapping with a miRBase entry and both miRNAs are overlapping with known miRNAs by at least 75%. (2) *Shifted known*: when the prediction is only partially overlapping with miRBase and only one miRNA is overlapping by at least 75% with a known miRNA. (3) *One annotated*: when the prediction is overlapping with a miRBase entry, but only one miRNA is annotated for that entry and this one is overlapping by at least 75%. (4) *Dissimilar overlapping*: when the prediction is overlapping with a miRBase entry, but the miRNAs are not overlapping with the annotated ones. (5) *Half novel*: when the prediction is not overlapping with any miRBase entry, but contains at least 75% of one known miRNA. (6) *Novel*: when the prediction is not overlapping with any miRBase entry and does not contain any known miRNA.

### Prediction flagging of other ncRNAs

In order to reduce the number of potential false positives, we map the predicted precursors to the Ensembl human non-coding RNA database (release 85) and to NONCODE 2016 (61) using BLAST+ (62) and flag them accordingly when matches are found. Further we map against the whole miRBase (v21) to highlight similar miRNAs in other species. Mappings are valid when over 90% of the aligned sequences overlap and at most one mismatch is present.

### Quantification of known and novel miRNAs, isomiRs and mutations

The quantification of known and novel miRNAs is performed analogously to miRDeep2. Reads are mapped against the precursors using Bowtie while allowing one mismatch. The counts are reported for all reads overlapping the annotated miRNAs in a window of up to two nucleotides upstream and five nucleotides downstream. IsomiRs are detected by mapping against the precursors using Bowtie while tolerating two mismatches. We allow up to two non-template additions to the 5′ and 3′ ends and up to one mismatch in between. We also allow a variability of two nucleotides at the 5′ end and of five nucleotides at the 3′ end per default. When detecting mutations, we focus on single nucleotide substitutions. The mapping and counting is performed the same way as the quantification, however miRNAs with mutations are explicitly counted.

### Exogenous read mapping

We map non-human reads (all reads that did not align to the human genome with at most one mismatch) to all 7556 bacteria and 7026 virus sequences of NCBI RefSeq (28) release 74 and report the number of perfectly mapping reads. Reads mapping to bacteria or viruses can indicate exogenous miRNAs, but also reagent contamination or diseases such as sepsis.

### Motif detection

Recently five miRNA motifs have been reported, namely the UG, UGU/GUG, CNNC (25), GHG (26) and GGAC (27) motif. We report for each prediction the present motifs, allowing matching up to two nucleotides upstream or downstream of the expected motif position.

### Usability

To analyze NGS miRNA samples with miRMaster, the user needs to provide sequencing files in FASTQ format (uncompressed or gzip compressed) without barcode sequence and the 3′ adapter used in the library preparation. After clicking on the ‘Launch experiment’ button on the homepage or in the navigation bar, the user will be guided through three steps. During the first one, one should name the experiment and also optionally provide an e-mail address to receive a notification as soon as the analysis of the uploaded samples is done. During the second step the user needs to specify the used 3′ adapter and has the opportunity to fine-tune the parameters of the analysis. The third step consists of the upload of the sequencing files. If the samples stem from multiple cohorts, groups can be specified by either clicking on the ‘Add second group’ button or by uploading a tab separated sample-to-group file. Once the files are chosen and the user has clicked the ‘Launch’ button, the data will be preprocessed and sent to the server. The preprocessing progress is shown directly on the web page whereas the server progress can be followed in real time by clicking the ‘Follow’ button. This will open the experiment status page in a new tab, where the user will be able to track the progress of the analysis of all uploaded samples. Real-time web reports are provided for each sample that has been uploaded, allowing to directly inspect the data. These reports provide information on the preprocessing, mapping, quantification and prediction steps. As soon as all samples have been analyzed, the results can be downloaded and an overall web-report is created with a link to it on the top of the status page.

### Validation using custom microarray

To perform a first pass iteration and to minimize the risk of false positives due to either NGS artifacts or low sample quality containing many degraded RNAs we designed a custom microarray containing all human miRNAs from the miRBase, the miRNAs from the study by Londin *et al*. (30) as well as over 5000 miRNAs from the present study. Among our predicted miRNAs we selected only those expressed in at least 50 samples which were not flagged as similar to other ncRNAs. The final microarray contained 11 866 miRNA candidates that have been measured each in 20 replicates (237 320 features per sample).

In order to measure the expression of the novel miRNAs in different human cells and tissues, we compiled a set of eight different human RNA samples: we purchased human total RNA samples from lung, brain, kidney, testis and heart tissues from Life Technologies (Cat. No. AM7968, AM7962, AM7976, AM7972 and AM7966, respectively) and the human miRNA reference kit from Agilent Technologies (Cat. No. 750700), that represents a pool of several human tissues and cell lines. Furthermore, we used a PAX blood RNA pool and a plasma RNA pool. The PAX blood RNA pool comprised of 11 blood samples collected in PAX gene tubes and purified with PAXgene Blood miRNA Kit from Qiagen according to manufacturer's instructions. Blood samples derived from four lung cancer patients, two Alzheimer's Disease patients, two patients with Wilms Tumor, and three healthy donors. The plasma RNA pool comprised of 10 plasma samples from healthy donors and was isolated using miRNeasy Serum/Plasma Kit after manufacturers recommendation with minor adaptations. To ensure sufficient RNA precipitation, we added 1 μl 20 mg/ml glycogen (Invitrogen) in the precipitation step. RNA concentration was measured using Nanodrop (ThermoFisher). RNA quality was assessed using Agilent Bioanalyzer Nano kit (for all tissue derived RNAs) or Small RNA kit (for the plasma sample).

The expression of 11 866 miRNAs and miRNA candidates was determined using the customized Agilent human miRNA microarrays. As input we used 100 ng total RNA as measured in Nanodrop for all tissue derived RNAs, and 1 ng miRNA as measured using Bioanalyzer Small RNA chip for the plasma sample. Using Agilent miRNA Complete Labeling and Hyb Kit after manufacturer's instructions, RNAs were dephosphorylated and labeled with Cy3-pCp. Labeled RNAs were hybridized to the custom microarrays for exactly 20 hours at 55°C. After hybridization, arrays were washed for 5 min in each Gene Expression Wash Buffer 1 (room temperature) and 2 (37°C). Subsequently, arrays were dried and scanned in an Agilent microarray scanner (G2505C). Expression data was extracted using Agilent feature extraction software. Downstream processing of signals has been carried out with R (version 3.2.4). Specifically, for clustering the expression intensities hierarchical clustering using the Euclidean distance has been performed as implemented in the Heatplus package.

To enable other researchers to repeat the experiments and to perform measurements on own samples, the microarrays that can be used analogously to standard Agilent microarrays using the Agilent protocols and SureScan platform, will be distributed by Hummingbird Diagnostics (Heidelberg, Germany) in three versions: human-mirna-candidate(full) containing all miRNA candidates from this study; mirna-candidate(detected) containing all miRNAs positive in any experiment of this study; mirna-candidate(blood) containing all miRNAs that have been detected in blood or serum.

## RESULTS AND DISCUSSION

**Figure 1.**
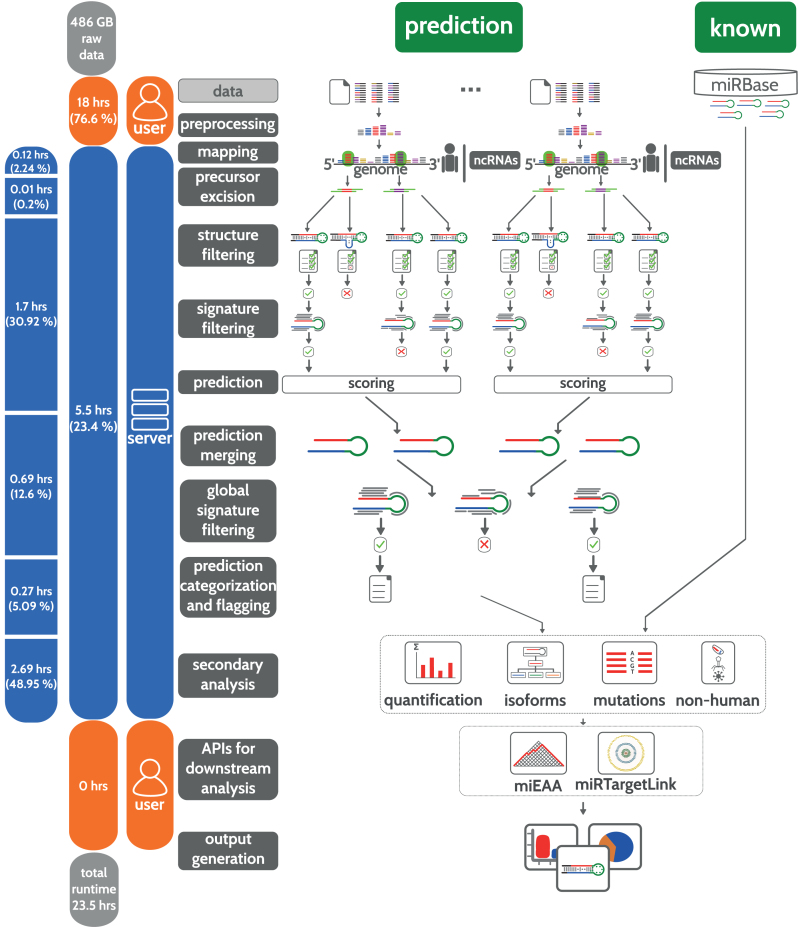
Schematic workflow of miRMaster. The bar at the left shows the runtime impact of each step. Steps performed by the user are shown in orange and steps performed by the server in blue.

The aim in developing miRMaster (www.ccb.uni-saarland.de/mirmaster) was to implement a comprehensive tool for the analysis of miRNA NGS data sets. Starting from raw or compressed FASTQ files with billions of reads and gigabytes of data, miRMaster allows a wide variety of miRNA analyses. The complete workflow is described in detail in the Methods section and sketched in Figure 1. A brief description on the usability of miRMaster is available in the Methods section.

In the following, we first focus on the performance of the novel algorithm for the prediction of new miRNAs. In total, we investigated 1097 miRNA NGS data sets containing 15 billion reads within a 486 GB file size and compare the miRMaster results – in terms of performance and runtime—to those of miRDeep2 using the same data sets. We next provide a detailed description of the different components of our miRNA NGS analysis framework and their application to the above-mentioned data set. Then we report a coarse description of the human miRNome by predicting small RNAs from 1836 data sets with 20 billion reads. Finally, we analyze the expression of potential miRNA candidates using custom microarrays.

### Evaluation of miRNA features

In contrast to most other comparable tools, our miRNA prediction relies on a broad set of features that are derived both from precursor sequences and from their mature forms. These features are considered as weak learners as each feature has a limited impact on the overall decision to classify or declassify a new miRNA as true miRNA. The feature set consists of 216 single features including nucleotide composition, secondary structure and others (the full list is available in Supplementary Table S1). To gain first insight into the discrimination power of single features we derived a positive miRNA precursor set from early miRBase (63) versions and from targets with strong experimental evidence in miRTarBase (42) (487 precursors), as well as a negative miRNA precursor set from various sources (12 384 negative precursors). A detailed explanation on the creation of these sets can be found in the Methods section (the sequences and locations of both sets are shown in Supplementary Table S2). We calculated the significance of all features by comparing both sets via Wilcoxon rank-sum tests. The performance of the 216 features is listed in Supplementary Table S1. The smallest significance value (10^−219^) was calculated for the minimum free energy index 1. Following adjustment for multiple testing, 158 of the 216 features remained significant (*P* < 0.05). Since our analysis pipeline is designed to support the evaluation of large data collections of up to several thousand samples, performance in runtime of feature calculation is of importance. We grouped all features in three different runtime categories with the fastest category containing features with 10,000-fold decreased runtime as compared to the slowest features. Supplementary Figure S1 shows the negative decadic logarithm of the *P*-values for features in the three categories. Since the two fast categories already contained 54 and 86 significant features, respectively, we evaluated their combined information content for predicting miRNAs. We derived classifiers not only from the complete feature set, but also from the fast features set only. Prior to classifying miRNAs based on the features we evaluated the redundancy of the features selected. As shown in the correlation heat map in Supplementary Figure S2 many of the features were redundant.

### Classification of precursors

For combining the predictive power of the weak learners we applied different feature selection and classification approaches. We selected a large variety of classifier and feature selection approaches, since there is no ‘one size fits all’ approach and our goal was to build a model that performs best on our datasets. Each of the tested classifiers and feature selection approaches have their strengths and weaknesses (e.g. SVMs with different kernels are suitable for different kinds of separation spaces). Since several single features show low discriminatory power (Supplementary Figure S1) and many features are correlated to each other (Supplementary Figure S2) it is important to define feature subsets that allow to classify or declassify a new miRNA precursor as true precursor. Different scaling and feature selection methods can have substantial effects on the used classifier. Therefore, we performed an exhaustive analysis of all combinations. We evaluated 130 105 different combinations of feature selection and classifiers using repeated stratified 5-fold cross validation. Even with the cross-validation, the evaluation of so many different classification attempts may lead to overoptimistic results. To address this problem, we performed permutation tests. The evaluation of the key performance criteria in Table 1 shows that almost all classifications were highly accurate. The area under the receiver operating characteristic curve (ROC AUC) highlights median performance of 99%, with the 90% quantile of all approaches being at 99.5% and more impressively the 10% quantile being at 95.8%. In consequence, 90% of all 130 105 tested classifiers had an AUC exceeding 95.8%.

**Table 1.** Cross validation performance

	Specificity	Sensitivity	Accuracy	NPV	Precision	ROC AUC	F_0.5_
Median	99.78%	70.62%	98.61%	98.90%	91.37%	98.98%	85.10%
90% quantile	99.91%	82.35%	99.18%	99.34%	95.61%	99.50%	91.81%
10% quantile	99.44%	45.17%	97.41%	97.97%	73.60%	95.85%	64.80%
AdaBoost (all features)	99.98%	86.85%	99.51%	99.51%	99.54%	99.58%	96.71%
AdaBoost (fast features)	99.98%	83.37%	99.38%	99.38%	99.26%	99.39%	95.60%

For both, the complete and the fast feature set AdaBoost outperformed the other models with an AUC of 99.6%, a specificity of 99.9% and a sensitivity of 86.9% for the complete feature set, and an AUC of 99.4%, a specificity of 99.9% and a sensitivity of 83.4% for the fast feature set. The selected AdaBoost classifier by itself selects only features known to improve the prediction and is therefore well suited for our broad set of features. This comparison demonstrates that the performance of the fast feature set is only marginally weaker than the performance of the full feature set. Nonetheless, we evaluated the performance of these two models and carried out stratified 5-fold cross-validation with 1000 repetitions each. The same approach was done with 1000 permutation tests each. As shown in Supplementary Figure S3, random test performance did not compare to the true performance in any of the cases and cross validation performance was stable and good in all cases. This further suggests that the composition of the cross-validation splits plays no major role for the model performance. In addition to the cross-validation performance we evaluated our model with the fast feature set on two independent test sets. A description of the independent test sets can be found in the Materials and Methods section. The first test set was composed of 129 human precursors and 28 human pseudo precursors. On this set our model reached a sensitivity of 82.9% and a specificity of 100%. The second test set contained 350 mouse precursors and 56 mouse pseudo precursors and resulted in a sensitivity of 81.4% and a specificity of 98.2%.

### Evaluation of prediction from 1097 miRNA NGS samples

Having evaluated the performance of our classifier on the positive and negative training set we applied the models to 1097 in-house data sets (33–39). These contain 15 billion reads in a total file size of 486GB (see Table 2). Again, we first compared the fast feature set versus the complete feature set. The prediction was carried out for each sample individually. They were then merged and filtered according to their global read signature. The differences between the models regarding known miRNAs were minimal with both models discovering 900 precursors, while 55 additional were uniquely found in the fast model opposed to 34 in the full model, as shown in Supplementary Figure S4. As for the novel miRNAs both models discovered 10 651 precursors. We then compared the unique predictions of both models in regard to their mean probability, novoMiRank score and the number of samples they were predicted in. We found that their mean scores and the mean number of samples they were predicted in were very similar (score of 1.18 for the complete model, 1.19 for the fast one; predicted in 7.5 samples for the complete and 7.6 for the fast model). However, we noticed also that for both sets the majority of the differing predictions were near the decision boundary with a mean probability below 60% (in contrast to an average of 70% for the common set), meaning that these predictions were among the less likely precursor miRNA candidates. Therefore, since both models performed very similarly, except for the less likely candidates, we further focused on the fast model, due to its runtime advantage.

**Table 2.** Composition of all 1836 NGS samples

Source / Description	#Samples	#Reads	Compressed File Size
CNS lymphoma patients and controls (in-house)	44	884 Mn	25GB
Alzheimer patients and controls (in-house)	203	3.4 Bn	114GB
Cardiovascular disease patients and controls (in-house)	485	6.9 Bn	205GB
Multiple sclerosis patients and controls (in-house)	217	1.2 Bn	44GB
Blood cell fractions from healthy donors (in-house)	148	3.3 Mn	98GB
GSE64142 (monocyte-derived dendritic cells upon bacterial infection)	116	1.4 Bn	43GB
GSE53080 (myocardium, plasma and serum in heart failure patients)	185	925 Mn	36GB
GSE49279 (adrenocortical tumors)	78	1.2 Bn	34GB
GSE45159 (adipose tissue)	360	786 Mn	24GB
**Sum**	**1836**	**20 Bn**	**623GB**

### Comparison between miRMaster and miRDeep2

To further evaluate the performance of miRMaster we compared its predictions with the predictions of miRDeep2, one of the central programs for miRNA discovery. In detail, we ran miRDeep2 with default parameters on our 1097 NGS samples and merged the overlapping precursors predicted by miRDeep2 by retaining the precursors predicted in most samples. The same procedure was applied for miRMaster. A more detailed description of the different analysis steps can be found in the Methods section.

**Figure 2.**
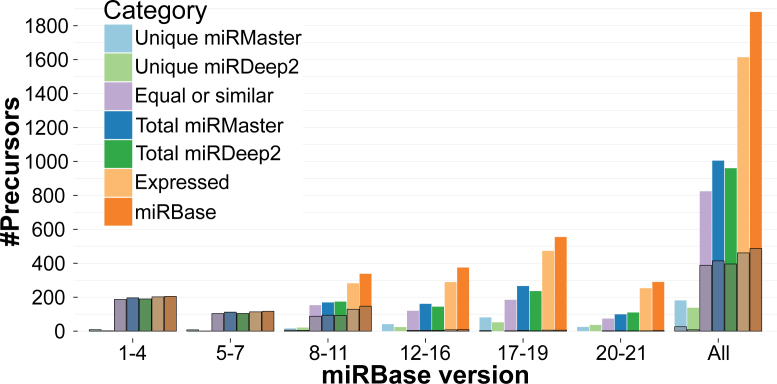
Distribution of recovered known miRBase precursors using miRMaster and miRDeep2. Predicted precursors are regarded as similar if they overlap by at least 90%. The black boxes show the number of precursors contained in the training set of miRMaster.

As shown in Figure 2, miRDeep2 recovered 59.5% of the known miRBase (version 21) precursors detected by quantification while miRMaster found 62.3% of them. Further, miRMaster consistently recovered more precursors from our training set than miRDeep2 (in total 414 versus 396). Specifically, 181 precursors were exclusively found by miRMaster and 138 by miRDeep2 as shown in Supplementary Table S3. Figure 2 shows that both tools perform especially well in earlier miRBase versions with both tools reporting nearly all precursors up to miRBase version 7. Precursor miRNAs exclusively detected by miRDeep2 are mainly found in later miRBase versions and contained only 7 precursors of miRNAs with strong experimental evidence for targets in miRTarBase. By contrast miRMaster detected 21 precursors in later miRBase versions with strong experimental evidence for targets in miRTarBase. These results might be biased since our models contain many more features and are trained using human high-confidence miRNAs on the one hand, and many miRNAs in later miRBase versions have already been reported by miRDeep2 on the other. Overall, the data suggest that our classifier identifies more known miRNAs and especially more of the strongly confident miRNAs.

To present a realistic comparison in runtime of miRMaster and miRDeep2, we measured execution time on the same infrastructure starting from pre-processed data. The computations were performed on a node with four AMD Opteron 6378 (4 × 16 cores totaling 64 cores) at 2.4 GHz and 512GB DDR3-RAM. MiRDeep2 required 102.5 h (4.4 days) without PDF generation (usually increases the runtime by 40% and produces reports for each known and predicted precursor). The respective steps of miRMaster required only 5.5 h which is a 19-fold decrease in runtime compared to miRDeep2. The difference is especially notable since miRMaster performed many additional analyses such as prediction of isoforms, variants in miRNAs and others. This difference in runtime is explained by the computed features and by different implementations. While miRDeep2 is implemented in Perl, miRMaster relies on a more efficient implementation in C++ for substantial parts of the program. One example is the precursor excision step, a reimplementation of the miRDeep2 Perl code in C++. This part of the program is roughly 40-fold faster in miRMaster as compared to miRDeep2.

A detailed break-down of the runtime in the different steps is presented in Supplementary Figure S5. The reads are mapped against miRBase and multiple other ncRNA databases (1.52% of the runtime) and to the human genome using Bowtie (56) (0.72% of the runtime). The afore mentioned precursor excision step requires 0.2% of the runtime. The following steps that are central for miRMaster include precursor segmentation, filtering, feature computation and prediction, altogether requiring 30.92% of the runtime. The predicted miRNA precursors from different samples are subsequently merged and filtered according to the read profiles of all samples (12.60% of the runtime). The following assignment to one of six categories ‘known’, ‘shifted known’, ‘one annotated’, ‘dissimilar overlapping’, ‘half novel’ or ‘novel’ requires 0.75% of the runtime. For the prediction flagging step, ncRNAs from Ensembl (57), lncRNAs from NONCODE (61) and known miRNAs from miRBase are mapped against the precursors (4.34% of the runtime). Finally, different secondary analyses are carried out on known and novel miRNAs, including quantification, which is again a reimplementation of miRDeep2, detection of isoforms and single base mutations. These steps, including the mapping of non-human reads to a collection of 7556 bacteria and 7026 viruses of NCBI RefSeq, permitting the detection of potential exogenous miRNAs, require in total 48.96% of the server runtime.

### Web-based analysis using miRMaster

With the development of miRMaster we aimed to provide a comprehensive web-based toolbox for an all-in-one miRNA analysis. In detail, the web-based tool has to (a) enable the analysis of HT-sequencing raw data without installing any software, even for data sets in the range of dozens of gigabytes; (b) perform the most common and further specialized analyses in an integrative manner; (c) return the results in a manner to be used for identifying interesting hits and for publication purposes by wet-lab scientists. These analyses are carried out in a fully integrated manner. From the raw data input (1097 compressed FASTQ files, 486GB) to final results for all calculations, miRMaster required 23.5 h. Data upload at client side was performed on an Intel Core i5–5200U Notebook with 12GB DDR3-RAM using Mozilla Firefox 48 and required most of the time (18 of the 23.5 h), while the analysis of pre-processed data took only 5.5 h. At client side, FASTQ files are first pre-processed (adapter trimming, quality filtering, read collapsing) and subsequently uploaded. The functionality is implemented in JavaScript such that no software has to be installed by the user. The runtime of this step may vary based on the equipment at user site and the bandwidth for data upload. Real world tests have demonstrated that studies including e.g. 50–100 samples can be evaluated in well below 5 h.

### Evaluation of variations in miRNAs by miRMaster

First, we investigated the mutation frequency. For each known miRNA of each of the 1097 samples we searched the number of single base mutations. To reduce a bias depending on the coverage we considered only miRNAs and their variants covered by at least 30 reads in 100 samples. Out of 2147 detected miRNAs 333 fulfilled the criteria. Supplementary Table S4 lists the mutations found in all miRNAs. Overall the largest number of variants was discovered for hsa-miR-486-5p, which is abundantly expressed across all samples with two precursors. However, for the majority of miRNAs the number of variants is low with most miRNAs having two or less variants (67.3%). For some miRNAs, such as hsa-miR-6131 the unmutated form was almost never detected and only variants with mutations at position 8 and 14 were found. Another example is hsa-miR-1260b with the most abundant form showing an A→G mutation at position 8 (Supplementary Figure S6). However, for most miRNAs (91.6%) the wildtype was most expressed. Our results suggest that only a small set of miRNAs is frequently affected by mutations e.g. due to RNA editing. The low number of mutations is to be expected, since mutations, especially in the seed region, are likely to highly affect the miRNA regulation network.

**Figure 3.**
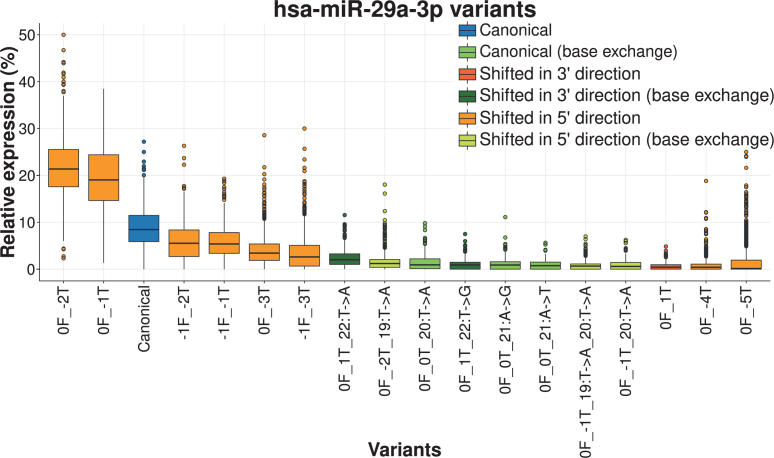
Isoform distribution of hsa-miR-29a-3p. Only variants appearing with an evidence of at least 30 reads in 100 samples are shown on the x-axis. Only reads occurring at least 30 times in a sample are shown for the relative expression to avoid large outlier due to low raw expression. Isoform notation: the number before F stands for the distance to the canonical 5′ end, in 5′-3′ direction (i.e. positive for trimmed, negative for extended); the number before the T stands for the distance to the canonical 3′ end (i.e. negative for trimmed, positive for extended). The canonical form is the third most frequent one and is highlighted in blue. Variants without base exchange are frequently shorter or shifted in the 5′ direction (orange), those with base exchanges match either the star/stop of the canonical miRNA (green) or are shifted slightly to the 5′ (light green) or 3′ (dark green) direction.

Next, we calculated for each known miRNA the number of isoforms, analogously to the steps performed for the detection of single base mutations. After applying the abovementioned filter criteria, we found 277 miRNAs isoforms that are listed in Supplementary Table S5. As for the mutated miRNAs we found the by far largest number of isoforms for hsa-miR-486-5p, which is highly expressed in blood. In consistence with the single base mutation results, the number of variants is low for the majority of miRNAs with most miRNAs (53.8%) showing four or less variants. For most miRNAs (71.5%) we detected the canonical form as annotated in miRBase. The miRNA with most variants and without canonical form was hsa-miR-107. As shown in Supplementary Figure S7, the most expressed form of hsa-miR-107 with a median of over 60% was trimmed by four nucleotides from the 3′ end, resulting in a miRNA with 19 nucleotides. Further, we frequently observed a lack of a dominating isoform over all samples, as for example for hsa-miR-29a-3p (Figure 3). This is consistent with the idea that isoform expression varies depending on the context, such as the cell type, time or population. Since the canonical form was most expressed in only 33.6% cases, isomiRs apparently play an essential role in miRNA function.

### Comprehensive version of the human miRNome

Currently, the total number of human miRNAs is controversially discussed. While miRBase currently contains 2588 human mature miRNAs (version 21), several studies propose even larger sets (e.g. Londin *et al*. (30), Backes *et al*. (11), Friedländer *et al*. (31), Jha *et al*. (32)). There exist two major challenges. First, the different miRNA sets are partially overlapping or contain miRNAs shifted only by few bases, adding a substantial redundancy. Second, the miRBase contains many false positive miRNAs, especially in later versions.

Using miRMaster we attempted to generate a coarse description of the human miRNome, i.e. we wanted to describe as many putative miRNA candidates as possible, being well aware that false positives are included (e.g. tRNA fragments, piRNAs or artifacts). This collection of potential candidates can be used to minimize further redundancy in upcoming high throughput studies.

**Figure 4.**
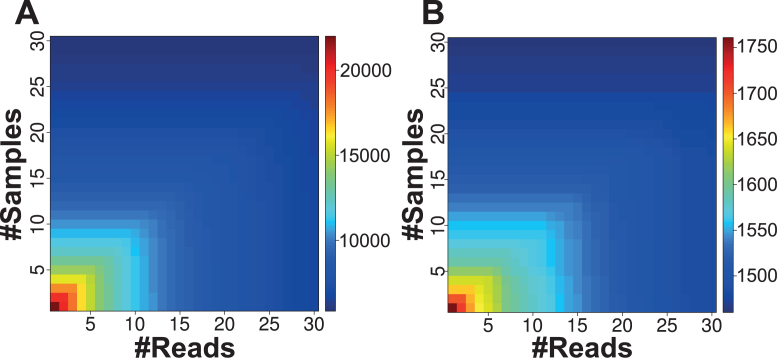
Distribution of the number of expressed precursors according to an evidence in a minimum number of samples and a total minimum number of reads. (**A**) The distribution of the number of expressed novel precursors. (**B**) The distribution of the number of known precursors.

Thus, in addition to our in-house NGS samples, we collected 739 samples from GEO (40), resulting in 1836 NGS samples (Table 2), and predicted novel miRNAs on those samples. The run resulted in 21 996 novel predicted miRNA precursors that are listed Supplementary Table S6. Those predictions can be inspected on the miRMaster webpage and downloaded as FASTA format. As shown in Figure 4A, most of the novel precursors were weakly expressed and in few samples. Considering only miRNAs with an expression in at least 30 samples reduced the number of predictions to 5845. As displayed in Figure 4B, the known precursors of miRBase (version 21) seem to be less affected by the augmenting number of samples or reads. Supplementary Figure S8 shows the number of expressed known and novel precursors according to their expression in multiple samples. The number of novel precursors decreases exponentially and faster than the known precursors with increasing number of required samples. This suggests that the majority of the commonly expressed miRNome is already known and that mainly tissue specific, time specific or other context specific miRNAs remain to be discovered.

Precursors of known and new miRNAs are evenly distributed on the positive and negative strands as shown in Supplementary Figure S9. The chromosomal distribution of known precursors largely matches with the distribution of the novel precursors as displayed in Supplementary Figure S10. In both cases, the least number of precursors can be found on chromosome Y. Chromosome 13, 18 and 21 harbor few known and novel precursors.

As for the number of motifs found in known and novel precursors with two annotated mature miRNAs, we found a slight enrichment of motifs in miRBase miRNAs (Supplementary Figure S11). A more fine-grained motif distribution is shown in Supplementary Figure S12.

**Figure 5.**
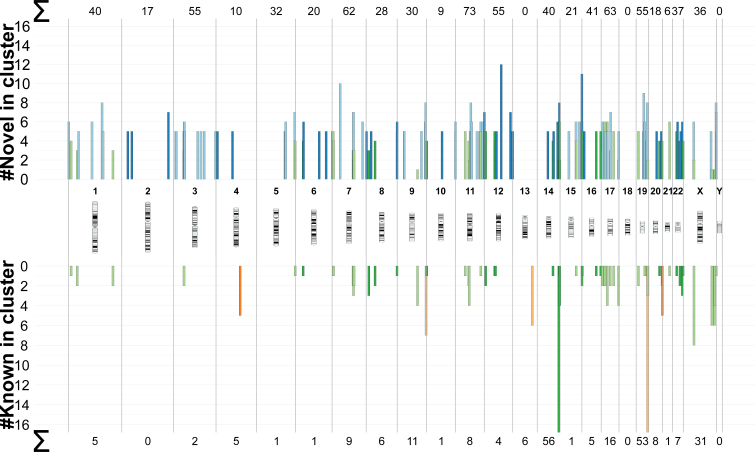
Distribution of the known and novel precursor clusters and their size on the human genome. Green clusters contain both novel and known precursors. Blue clusters contain only novel precursors and orange clusters contain only known precursors. The two known clusters on chromosome 14 and 19 (size 42 and 46) were trimmed for a better visualization. The sum of the number of novel or known precursors in all clusters of a chromosome with at least five members are shown on the top and bottom of the plot.

Since miRNAs often occur in genomic clusters, we also searched genomic regions that are enriched by novel miRNAs. Supplementary Table S7 lists the positions of clusters when allowing a distance of at most 10 kb between the middle position of known or novel precursors. The largest cluster was composed of 46 known precursors and spanned 96 kb on chromosome 19. The largest cluster that contained both known and novel precursors was found on chromosome 14 and contained 42 known and 2 novel precursors and spanned 45 kb. In total 3969 clusters contained either known or novel precursors. Of these, 3423 clusters contained exclusively novel precursors. Further, 455 clusters contained both known and novel precursors and 91 exclusively known precursors. Supplementary Figure S13A and B shows the number of clusters with at least two or five precursors on each chromosome. Most clusters (394) with a minimum size of 2 could be found in chromosome 1. When focusing on clusters with at least five members, the numbers decreased to 154 clusters, 93 of which contained exclusively novel precursors. Most clusters were observed on chromosome 11. Figure 5 shows the distribution of all clusters with five or more precursors over the human genome and demonstrates that many clusters contain both, known as well as novel precursors. The largest novel cluster with 12 precursors was found on chromosome 12.

To estimate how close our reported predictions might be to the coverage of the human miRNome, we performed predictions for different numbers of samples, each 10x randomly selected from our sample set. Supplementary Figure S14 shows the number of predictions according to the number of samples. We observe that the increase in number of predictions clearly exponentially diminishes with the number of samples. Since these predictions contain many false positives we expect the real part to be much smaller and the increase in predictions smaller as well. Therefore, we suggest that, at least for the tissues covered by our samples, we are close to the complete coverage of the human miRNome. We are aware and expect that the addition of samples of further tissue types or different conditions might add new candidates to our predicted set.

### Expression analysis of miRNA candidates using custom microarrays

**Figure 6.**
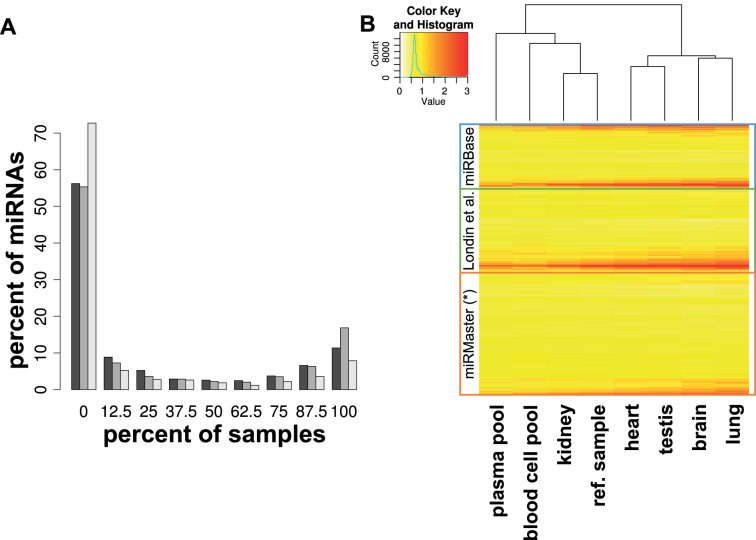
Expression of miRNA candidates on custom microarrays. (**A**) Distribution of the percentage of detected miRNAs in different samples. The colors correspond to the miRNAs of three studies: miRBase, dark gray; Londin *et al.*, medium grey; this study, light gray. (**B**) Heatmap of the logarithmized expression intensities of all miRNAs according to different tissues. For better visualization all expression values superior to 1000 were trimmed. The hierarchical clustering was performed with Euclidean distance.

To provide further evidence that a relevant fraction of the aforementioned mature miRNAs is not only due to NGS bias or other artifacts such as RNA degradation, we built a custom human microarray. This array contains all miRBase v21 miRNAs, the miRNAs from the study by Londin *et al*. (30) and the top ranking miRNAs from the present study. The final microarray contained 11 866 miRNA candidates that have been measured each in 20 replicates (237 320 features per sample). For the microarray hybridization, we selected tissues from our Tissue Atlas (64) that contained the most miRNAs and added body fluids harboring likewise many miRNAs (65). The set of samples included a pool of PAXGene blood samples, a pool of plasma samples, lung tissue, brain tissue, kidney tissue, testis tissue, heart tissue and a reference pool from Agilent. Since degraded RNA is known to affect the miRNA patterns, we ensured high-quality of the used RNA samples. The RIN values of the different specimens ranged between 7.5 and 9. For the three sets of miRNAs the percentage of positive miRNAs in the hybridization experiments is presented in Figure 6A. For 56% of miRBase miRNAs, 55% of miRNAs by Londin *et al*. and 73% of miRNAs from the present study no positive signal in any sample was observed. On the other extreme, 11%, 17% and 8% were respectively positive in all experiments. The larger fraction of miRNAs not detected in any sample in the third set can be explained by the fact that many of the high abundant markers were previously already detected while we selected the candidates from the not yet discovered and likely much less abundant fraction. Still the results presented above can contain false positives (e.g. reagent contamination or positive signals induced by fragmented other RNAs) and false negatives (e.g. since other tissues or samples may harbor the miRNAs negative in the presently used samples or that are negative because of the limit of detection of microarrays). The same pattern as described can be recovered from the cluster analysis of all miRNAs from the three sets in Figure 6B. The lower part of this heat map shows that especially context sensitive miRNAs are observed among the set of miRNAs candidates only reported by miRMaster. In sum, the data strongly suggest that miRNAs exist which are currently not annotated in the miRBase. These miRNAs deserve further validation. All miRNAs from this analysis are contained in Supplementary Table S8.

## CONCLUSIONS

The use of multiple web-based and standalone tools combined with different data formats makes the analysis of HT-seq miRNA data difficult, especially for wet-lab scientists. Therefore, we propose a web service that performs the most frequently requested applications directly from the raw FASTQ files. At the same time, experimental methods are advanced such that large-scale studies are feasible. Studies with many hundred or thousand samples are hard to be evaluated by current tools. Besides accuracy and specificity, runtime is among the most important criteria. Although miRMaster carries out a far greater number of analyses than other tools like miRDeep2, the running time of the miRMaster analysis was up to 20-fold faster. Of course, the precursor candidates predicted by miRMaster should in subsequent steps undergo a manual inspection and the selected ones be experimentally validated before calling them real miRNAs. A first validation step could be performed with our custom microarray followed by a more in depth validation of the detected interesting candidates using e.g. northern blotting. Applications such as target prediction, functional analysis and differential expression of known and novel miRNAs will in the future complete the portfolio of miRMaster.

### ACCESSION NUMBERS

NGS samples are available on GEO under the following accession numbers: GSE64142, GSE53080, GSE49279, GSE45159 and GSE46579.

## Supplementary Material

Supplementary Data
