# Supplementary material for: Web-based NGS data analysis using miRMaster: a large-scale meta-analysis of human miRNAs
Source: Nucleic Acids Res. 2017 Jul 12;45(15):8731–44. doi: 10.1093/nar/gkx595 (PMC5587802; doi:10.1093/nar/gkx595)
Supplement: Supplementary Data [file gkx595_Supp.zip › nar-01232-n-2017-File009.pdf]

Web-based NGS data analysis using miRMaster:

a large-scale meta-analysis of human miRNAs

Tobias Fehlmann[1*], Christina Backes[1], Mustafa Kahraman[1,2], Jan Haas[4,5,6], Nicole Ludwig[3], Andreas E. Posch[7], Maximilian L. Würstle[8], Matthias Hübenthal[9], Andre Franke[9], Benjamin Meder[4,5,6], Eckart Meese[3], Andreas Keller[1]

[1] Chair for Clinical Bioinformatics, Saarland University, Saarbrücken, Germany

[2] Hummingbird Diagnostics GmbH, Heidelberg, Germany

[3] Department of Human Genetics, Saarland University, Homburg, Germany

[4] Department of Internal Medicine III, University Hospital Heidelberg, Heidelberg, Germany

[5] German Center for Cardiovascular Research (DZHK), Heidelberg, Germany

[6] Klaus Tschira Institute for Integrative Computational Cardiology, Heidelberg, Germany

[7] Curetis GmbH, Holzgerlingen, Germany

[8] Siemens Healthcare GmbH, Strategy and Innovation, Erlangen, Germany

[9] IKMB, Christian Albrechts Universitat zu Kiel, Kiel, Germany

[*] Corresponding Author

Supplementary Information

Contents

Supplementary Figures

Figure S1: P-value distribution according to the runtime categories.

Figure S2: Correlation heatmap of all features using Pearson's correlation coefficient

Figure S3: Classification results of our two prediction models with 1000 5-fold cross-validation repetition and 1000 permutation tests

Figure S4: Comparison of the number of predicted precursors by our two models.

Figure S5: Relative runtime of all server based steps in the order of execution

Figure S6: Single base exchange distribution of hsa-miR-1260b

Figure S7: Isoform distribution of hsa-miR-107

Figure S8: Novel and known precursor distribution over the number of samples they are expressed in

Figure S9: Strand distribution of precursors

Figure S10: Chromosomal distribution of precursors

Figure S11: Distribution of the number of motifs found in precursors

Figure S12: Motif distribution in precursors

Figure S13: Distribution of the number of clusters found per chromosome

Figure S14: Distribution of the number of predictions per number of samples

Figure S15: Classifier generation process

Supplementary Tables

Table S1: List of all used features including a brief description, their runtime impact and their p-value. The p-value resulted from a two sided Wilcoxon rank-sum test after Benjamini-Hochberg adjustment (alpha=0.05) for multiple testing on our positive and negative datasets.

Table S2: List of all positive and negative precursors used in our training procedure.

Table S3: List of all precursors exclusively recovered by miRDeep2 and miRMaster on our 1,097 samples.

Table S4: List of all single base mutations found in known miRNAs expressed in our 1,097 samples. Only miRNAs and their variants covered by at least 30 reads in 100 samples were considered.

Table S5: List of all isoforms found for known miRNAs expressed in our 1,097 samples. Only miRNAs and their variants covered by at least 30 reads in 100 samples were considered.

Table S6: List of all predicted precursors on 1,836 NGS samples by miRMaster.

Table S7: List of the positions of all clusters of known and novel precursors when allowing a distance of at most 10 kb between the middle position of the precursors.

Table S8: List of all miRNA candidates present on the custom microarray.

Table S9: Different classification setups used for the classifier generation

Supplemental Figure 1: P-value distribution according to the runtime categories.

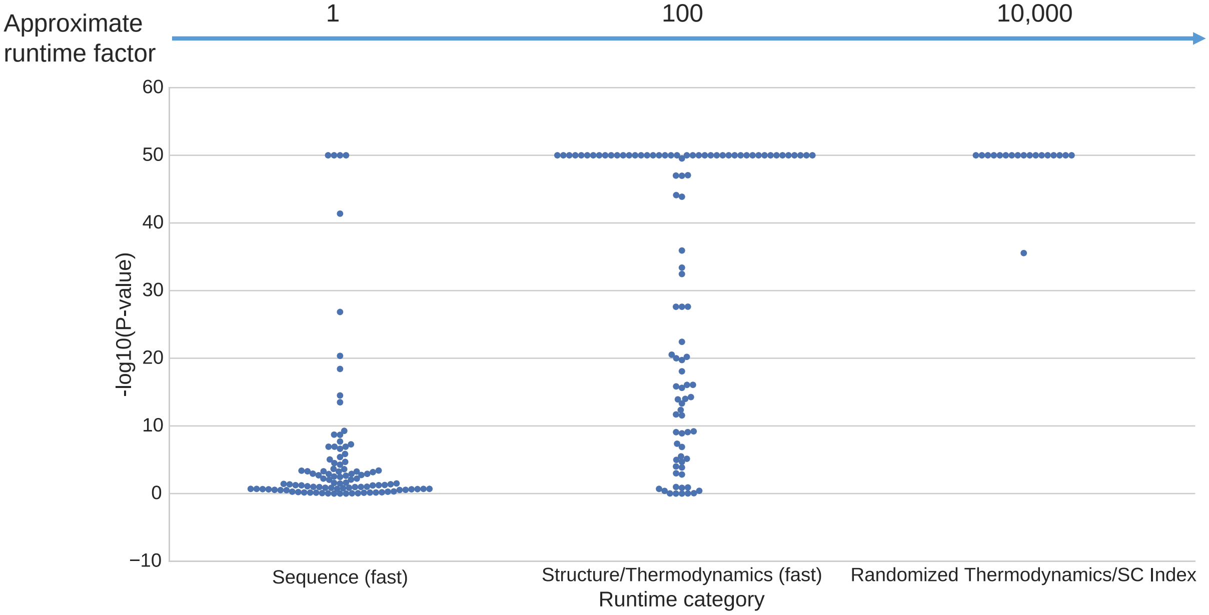

The P-values are computed with the two sided Wilcoxon rank-sum test and adjusted for multiple testing using the Benjamini-Hochberg procedure (alpha=0.05). Values were floored at 10^{-50} for a better visualization. The runtime factor for each category is approximate and relative to the sequence feature runtime.

Supplemental Figure 2: Correlation heatmap of all features using Pearson's correlation coefficient.

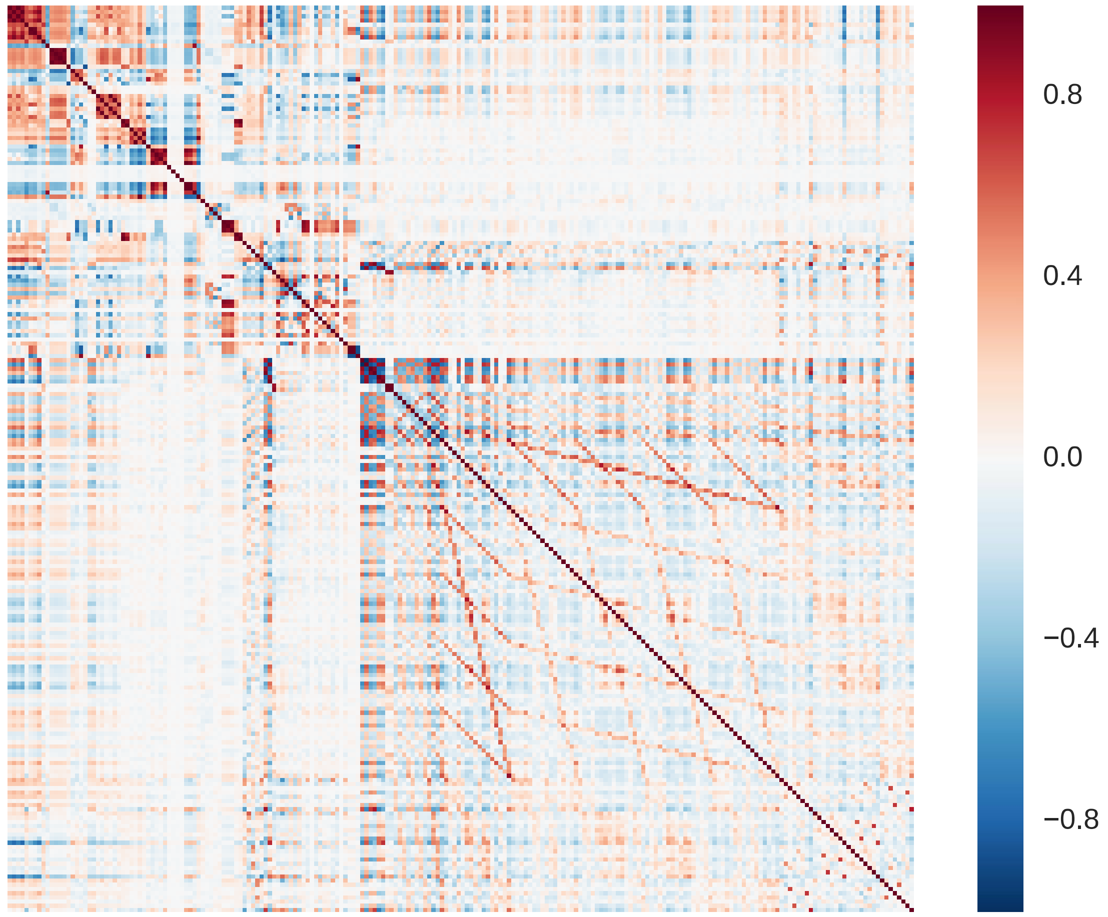

Supplemental Figure 3: Classification results of our two prediction models with 1000 5-fold cross-validation repetition and 1000 permutation tests.

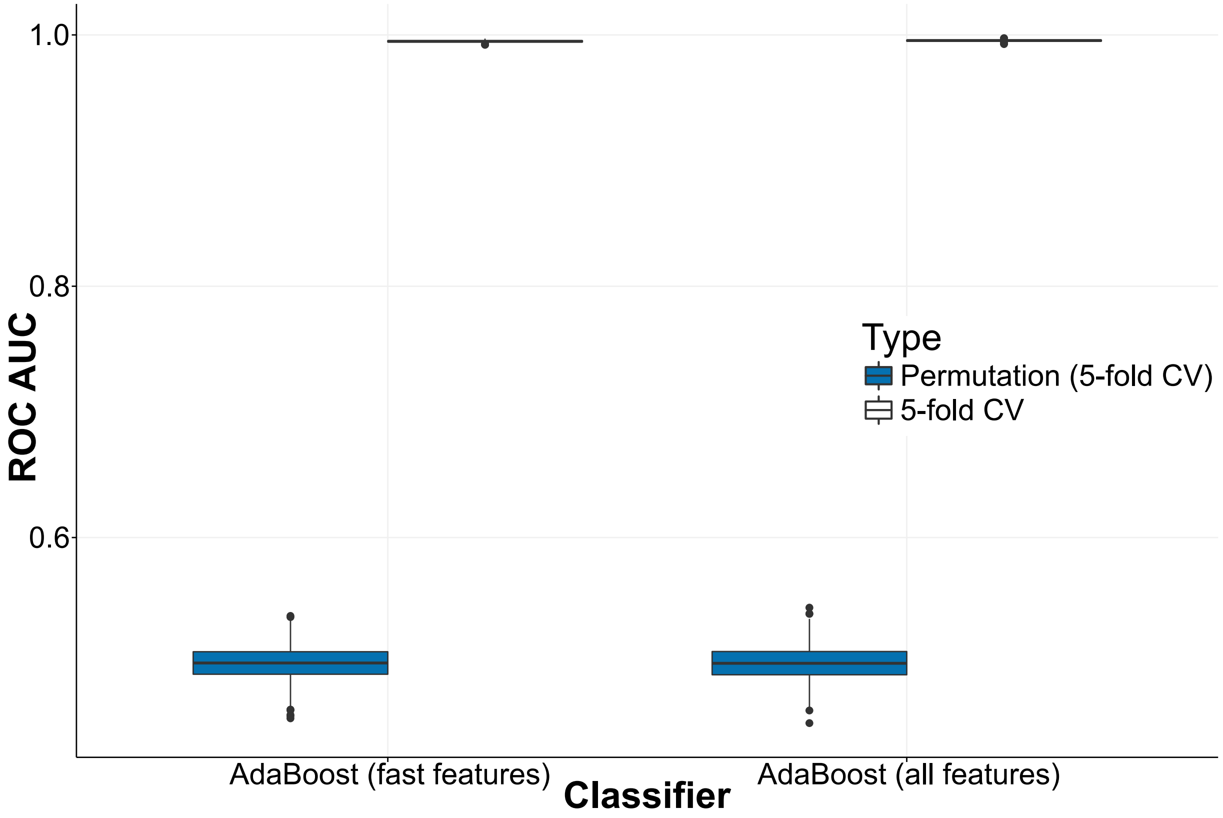

Supplemental Figure 4: Comparison of the number of predicted precursors by our two models.

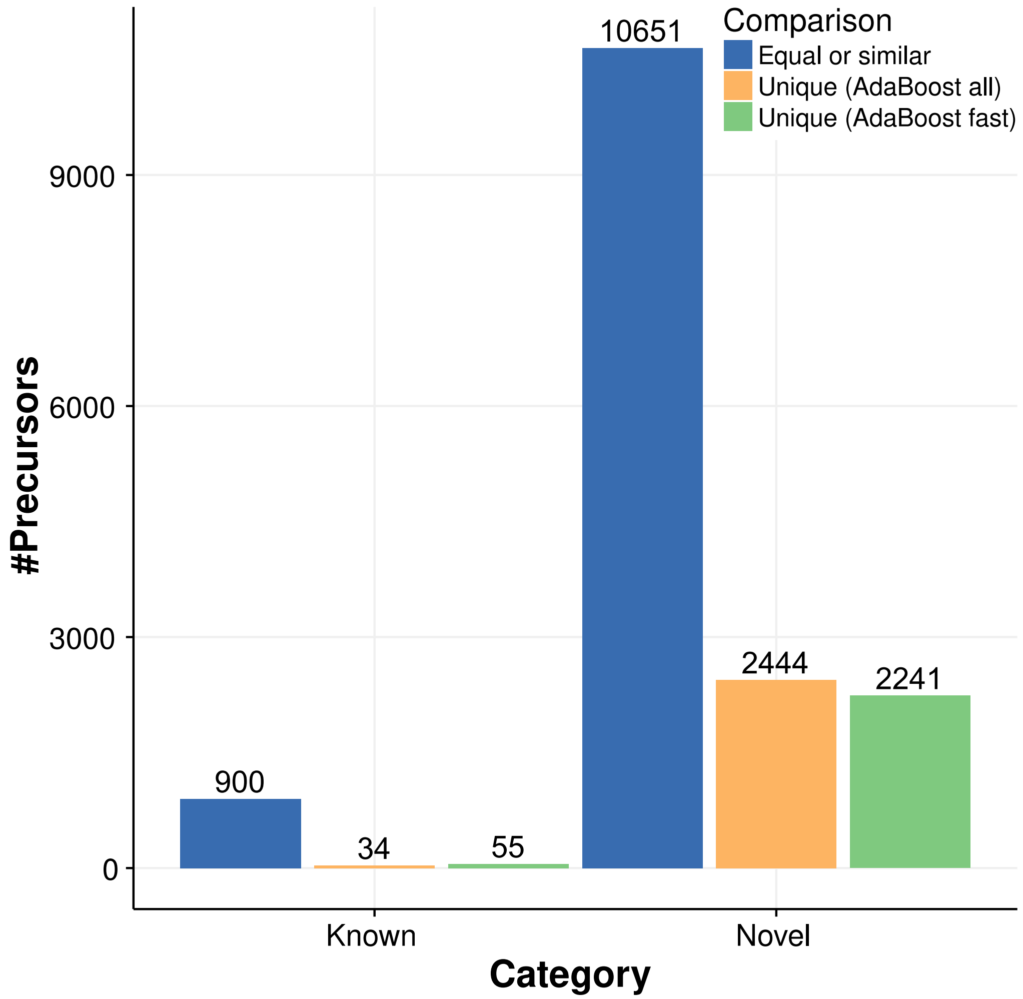

Predicted precursors are regarded as similar if they overlap by at least 90%.

Supplemental Figure 5: Relative runtime of all server based steps in the order of execution.

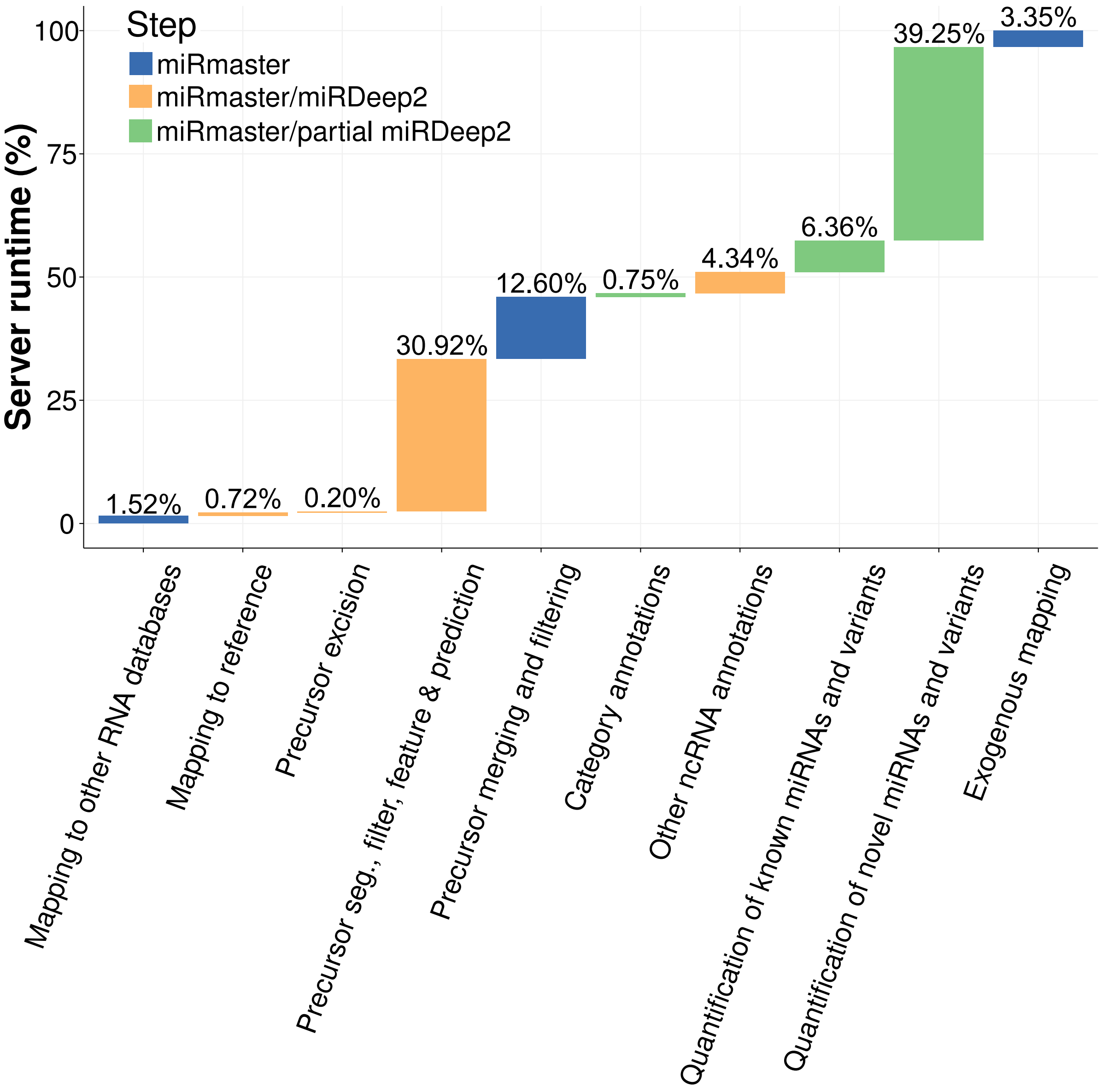

Supplemental Figure 6: Single base exchange distribution of hsa-miR-1260b.

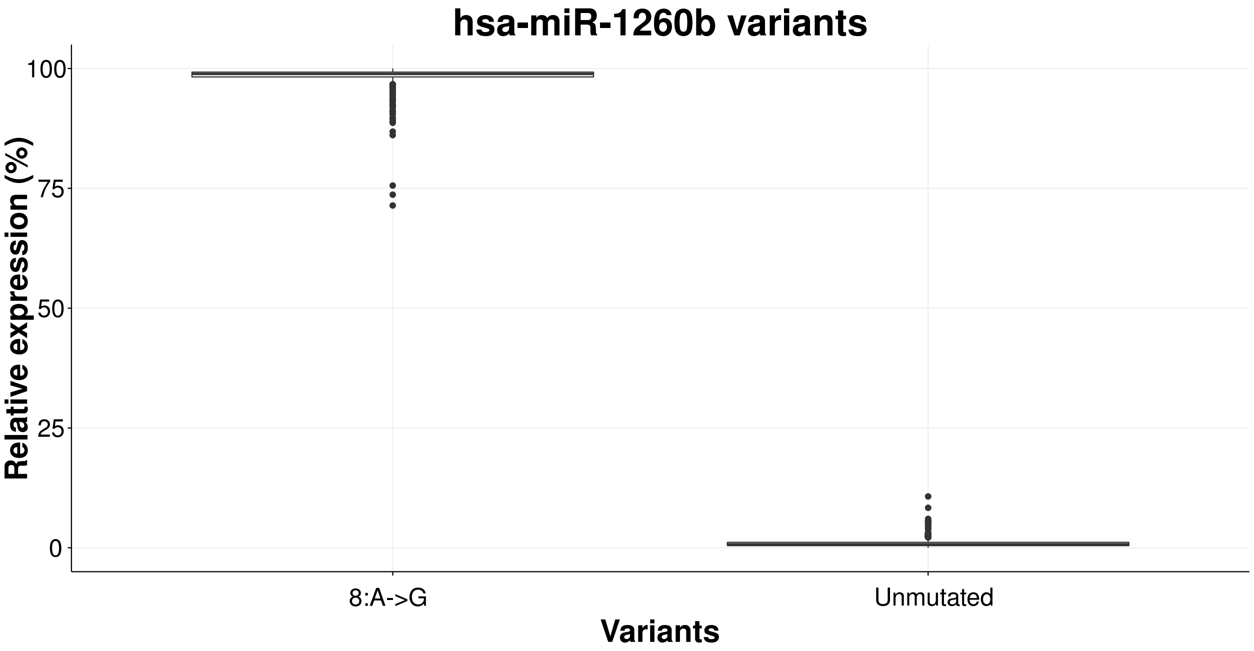

Only variants appearing with an evidence of at least 30 reads in 100 samples are shown on the x-axis. Only reads occurring at least 30 times in a sample are shown for the relative expression to avoid large outlier due to low raw expression.

Supplemental Figure 7: Isoform distribution of hsa-miR-107.

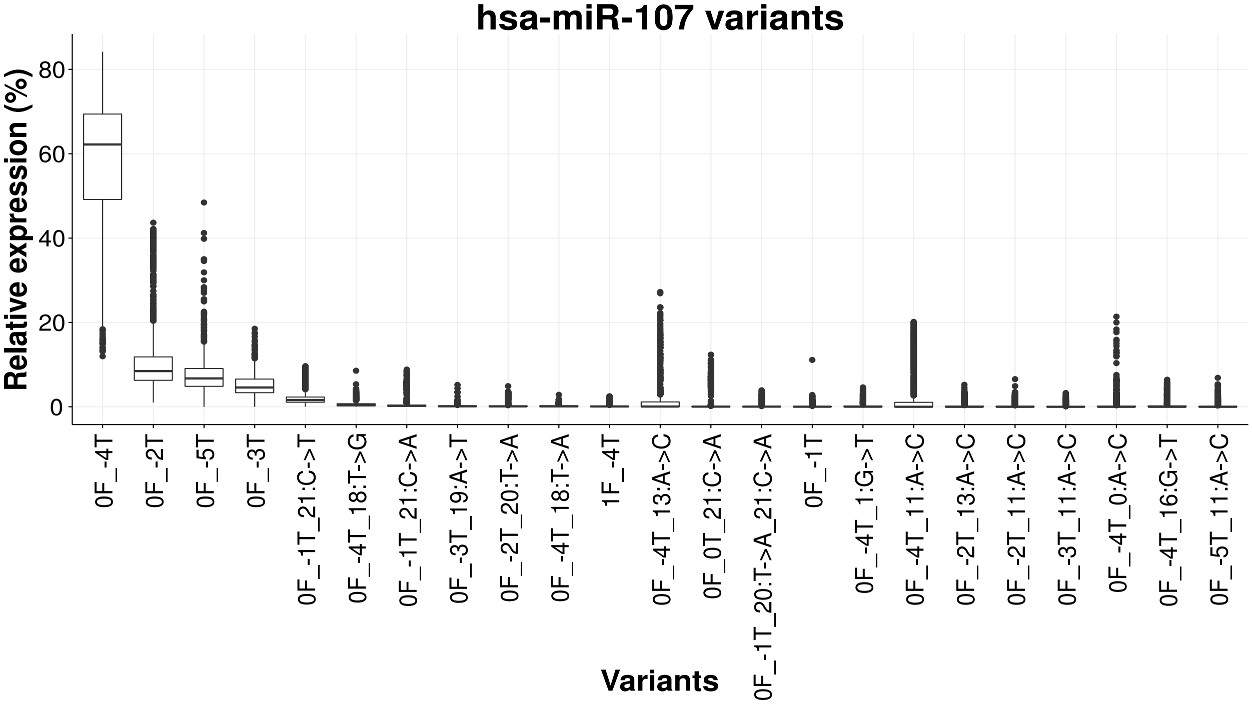

Only variants appearing with an evidence of at least 30 reads in 100 samples are shown on the x-axis. Only reads occurring at least 30 times in a sample are shown for the relative expression to avoid large outlier due to low raw expression. Isoform notation: the number before F stands for the distance to the canonical 5’ end, in 5’-3’ direction (i.e. positive for trimmed, negative for extended); the number before the T stands for the distance to the canonical 3’end (i.e. negative for trimmed, positive for extended).

Supplemental Figure 8: Novel and known precursor distribution over the number of samples they are expressed in.

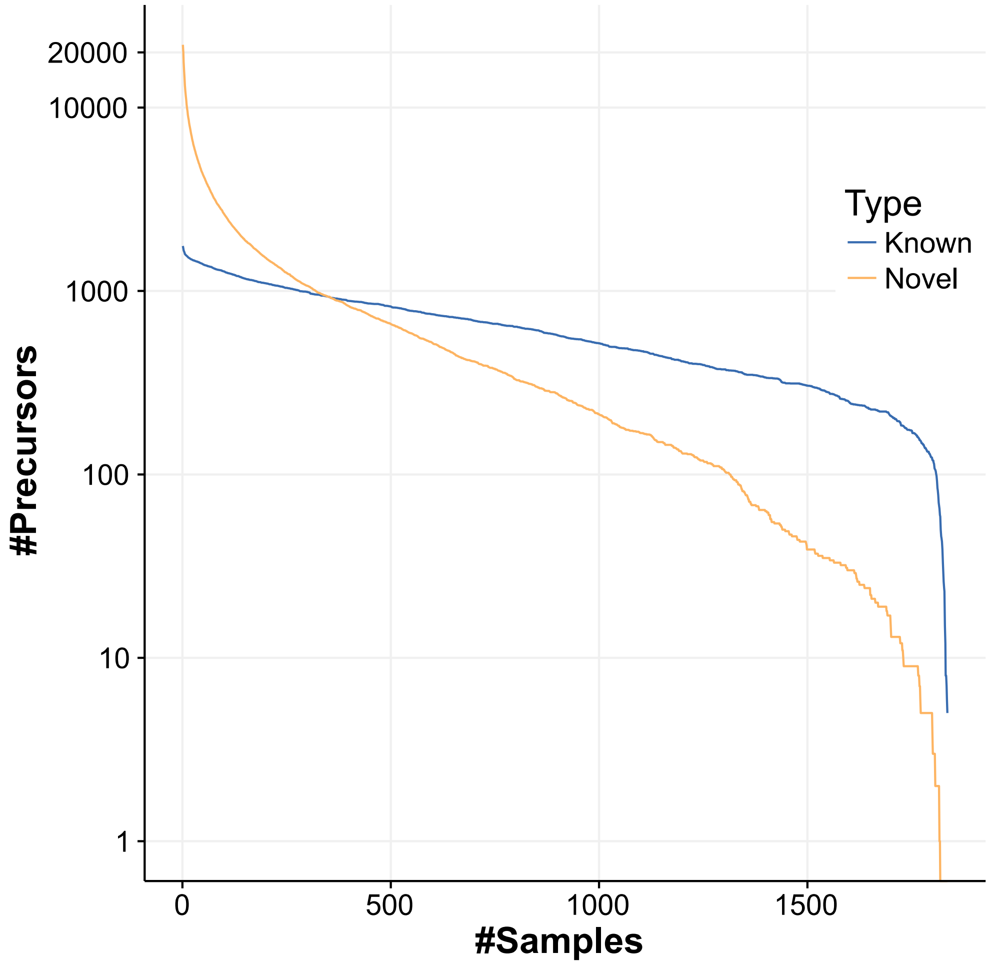

Supplemental Figure 9: Strand distribution of precursors.

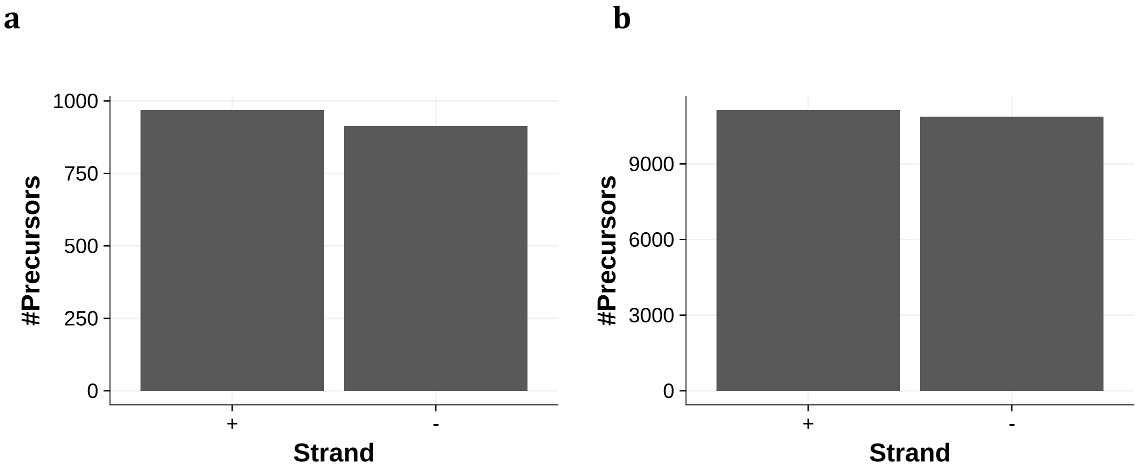

(a) Strand distribution of known precursors from miRBase v21. (b) Strand distribution of novel precursors.

Supplemental Figure 10: Chromosomal distribution of precursors.

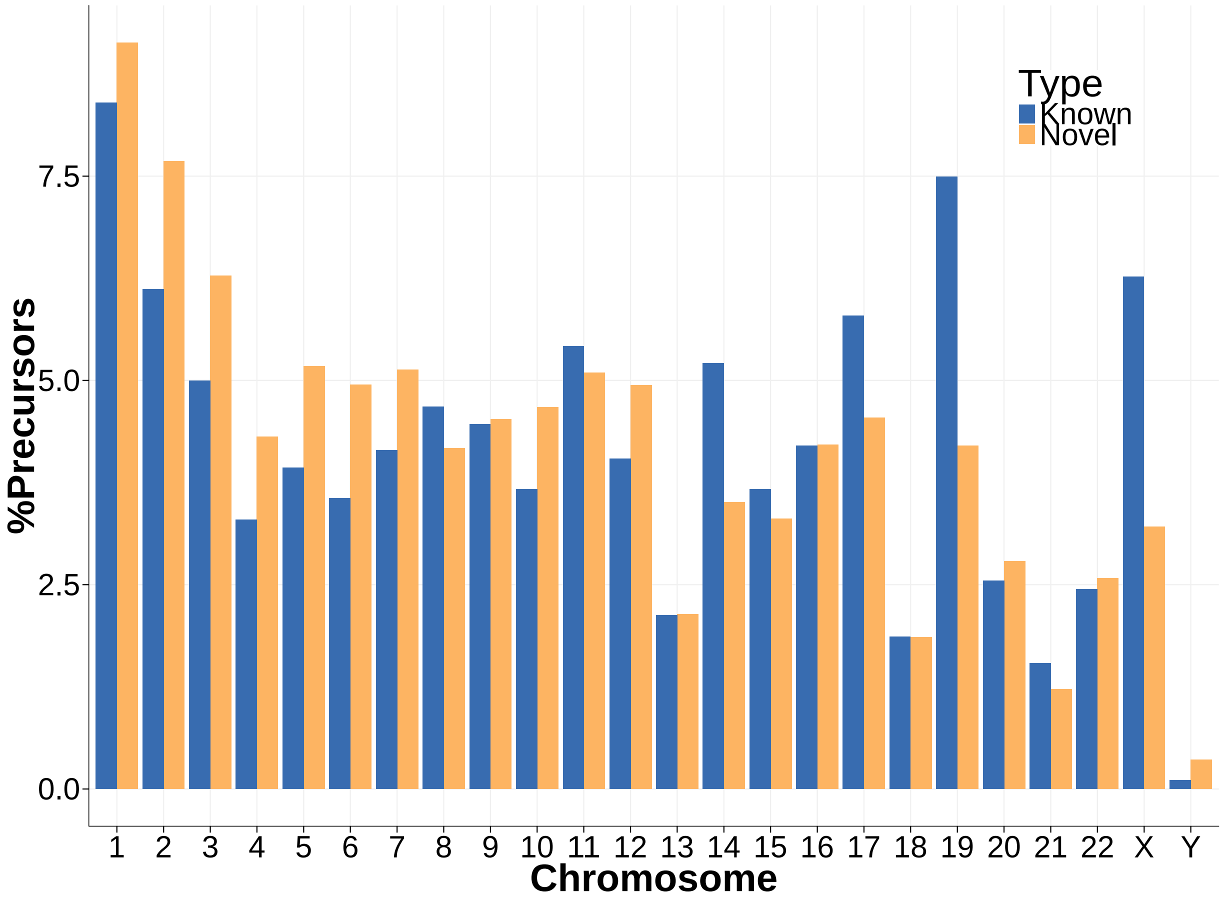

Chromosomal distribution of known and novel precursors in percent, relative to the respective total number.

Supplemental Figure 11: Distribution of the number of motifs found in precursors.

a **b**

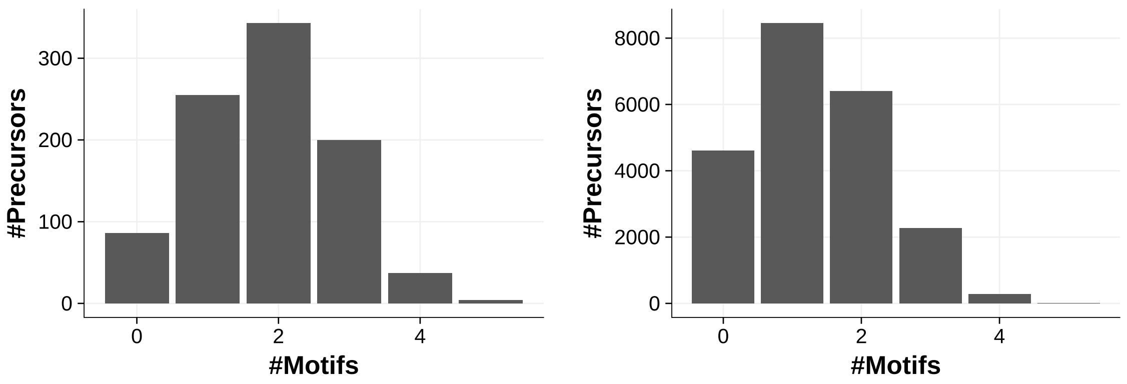

(a) Distribution of the number of motifs found in known precursors (only the ones with two annotated miRNAs) from miRBase v21. (b) Distribution of the number of motifs found in novel precursors.

Supplemental Figure 12: Motif distribution in precursors.

a

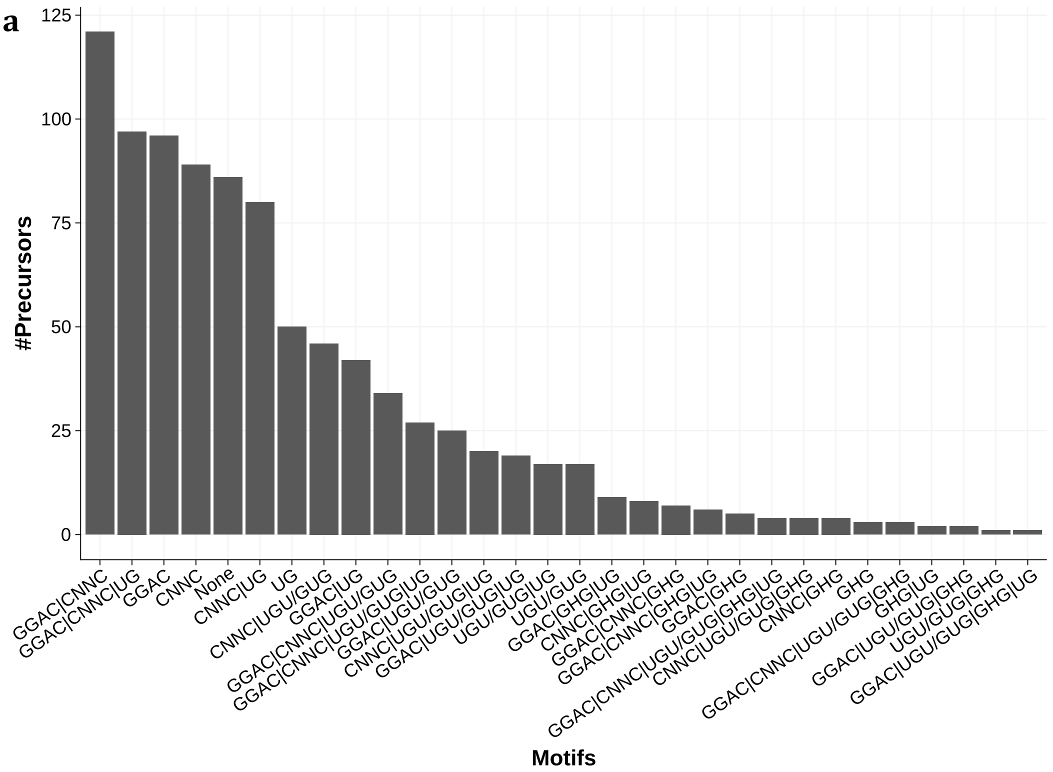

b

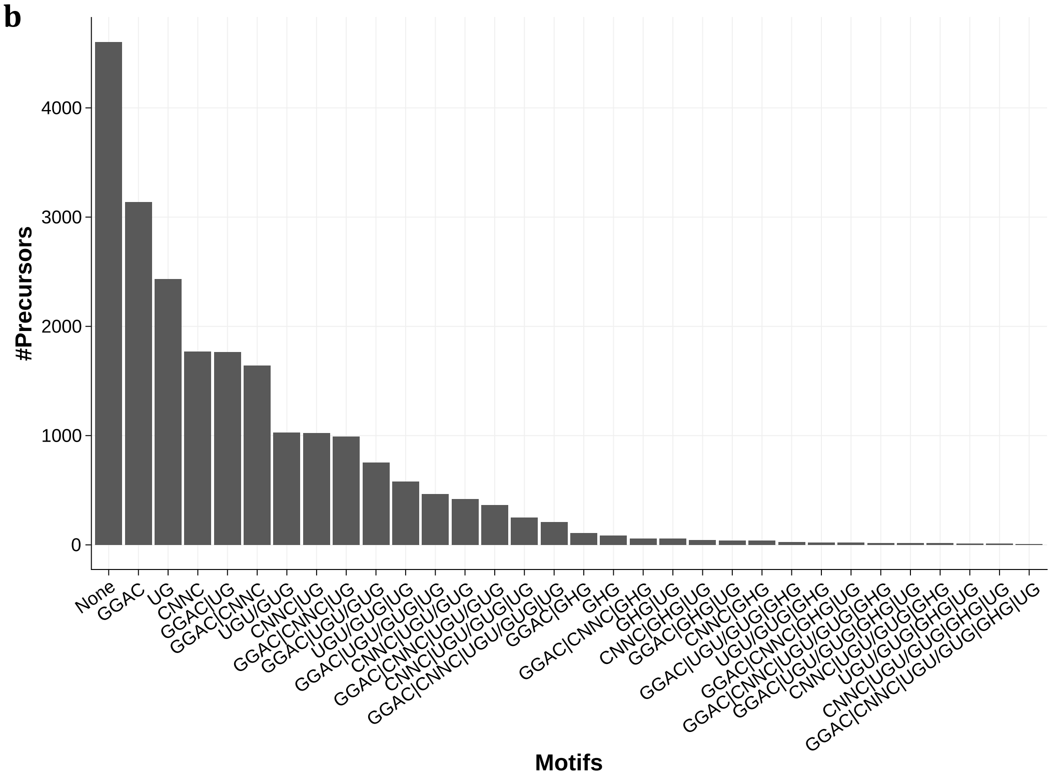

(a) Motif distribution in known precursors (only the ones with two annotated miRNAs) from miRBase v21. (b) Motif distribution in novel precursors.

Supplemental Figure 13: Distribution of the number of clusters found per chromosome.

a

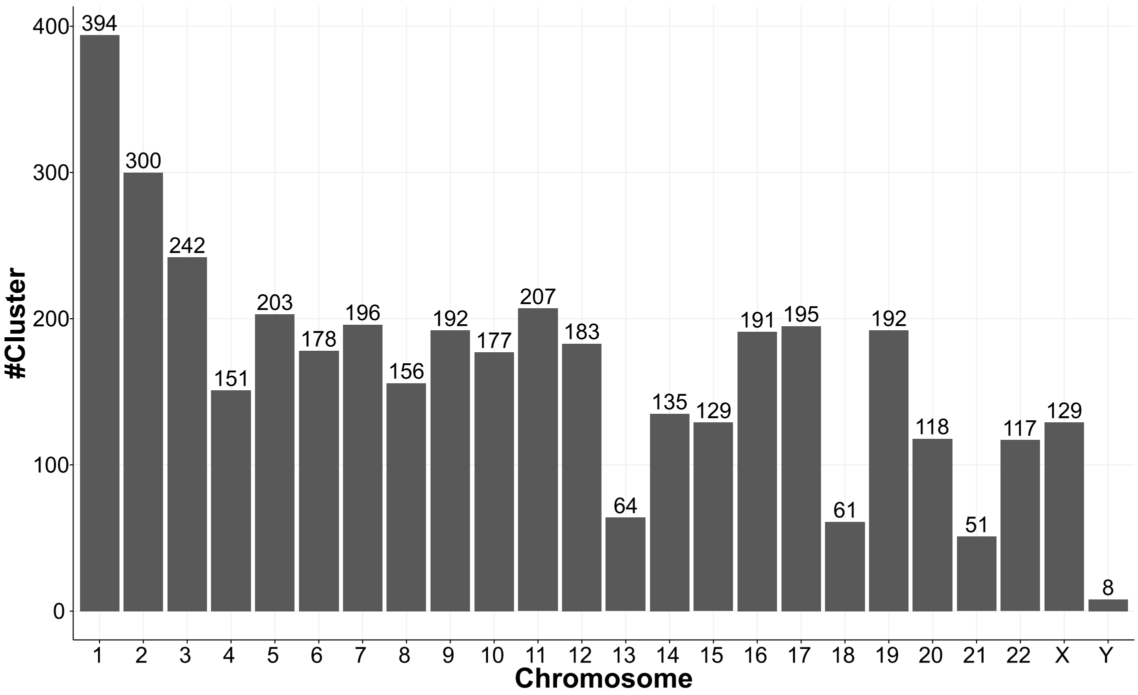

b

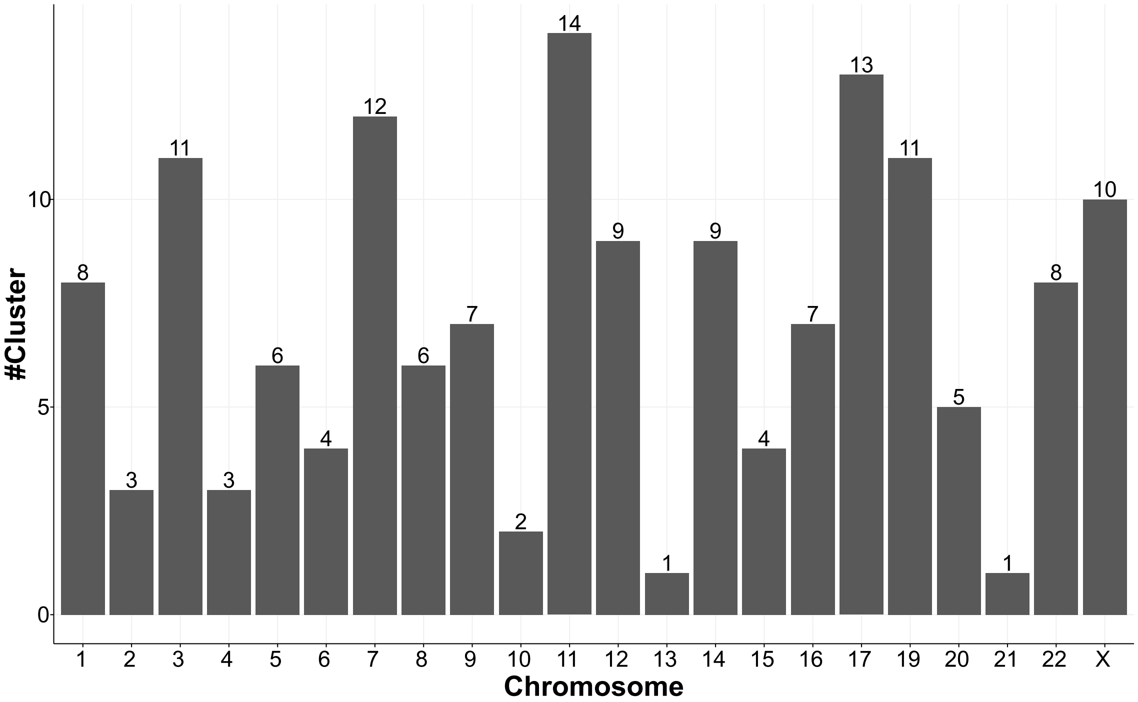

(a) Distribution of the number of clusters found per chromosome, with each cluster containing at least 2 precursors. (b) Distribution of the number of clusters found per chromosome, with each cluster containing at least 5 precursors.

Supplemental Figure 14: Distribution of the number of predictions per number of samples.

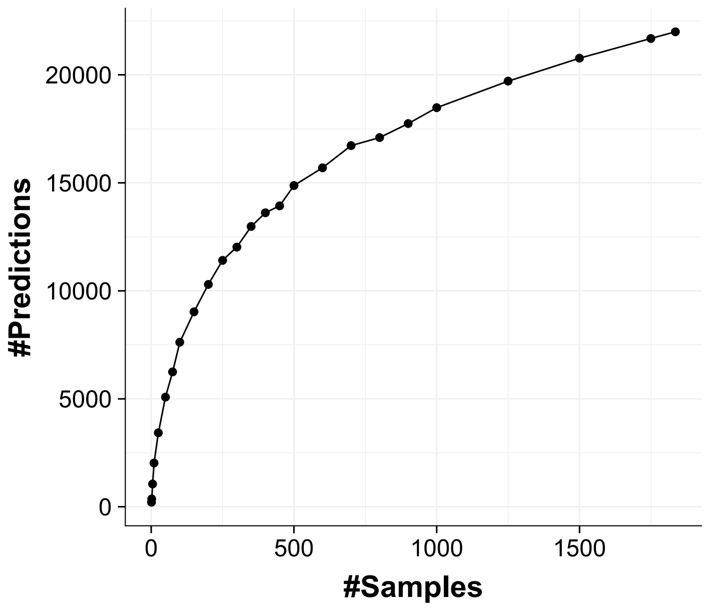

Mean number of predictions for different numbers of samples, each 10x randomly selected from the 1836 samples.

Supplemental Figure 15: Classifier generation process.

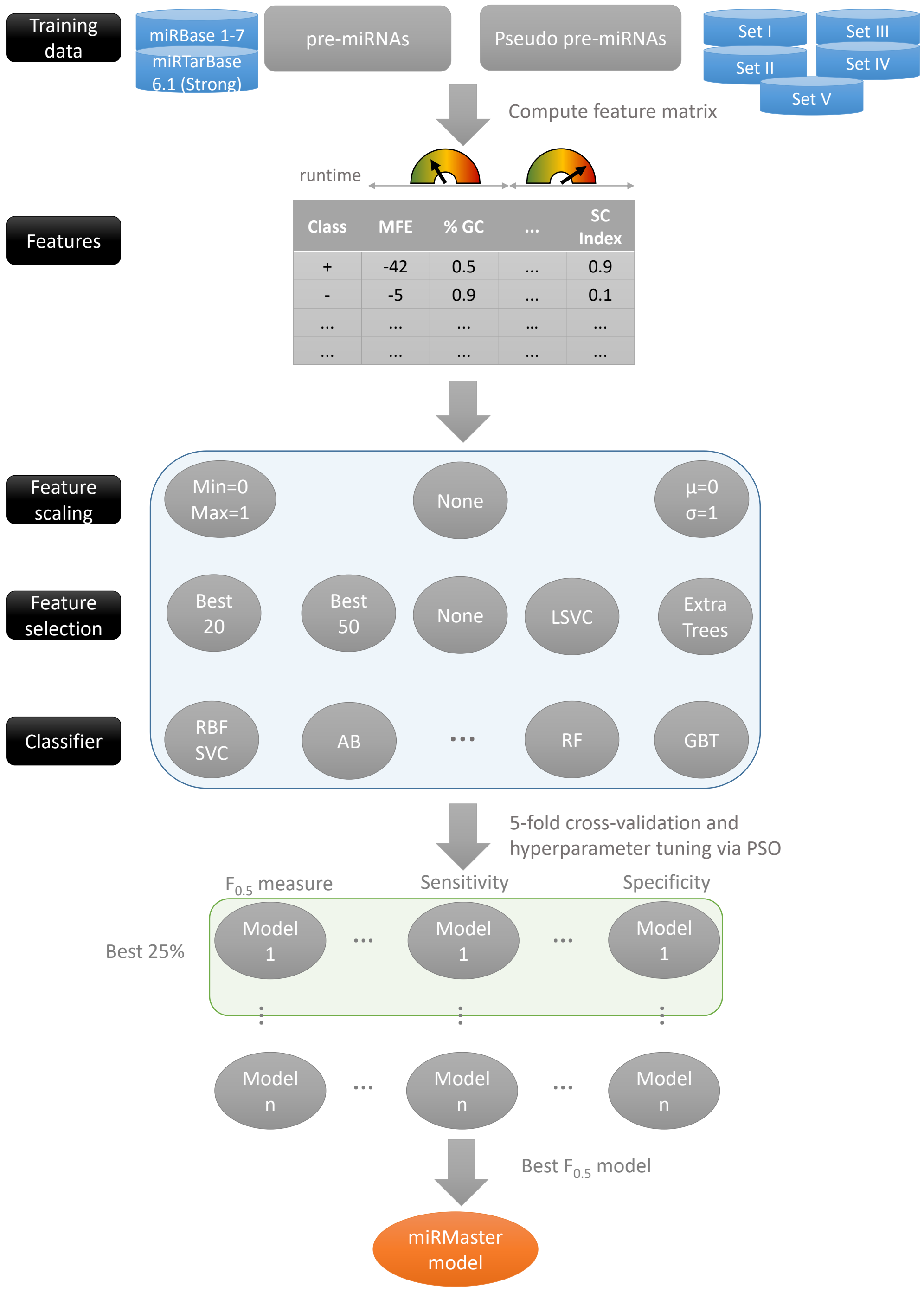

The features are computed for the positive and negative datasets. Then combinations of feature scaling, selection and classifier are used to train various models, each using 5-fold cross-validation. The hyperparameters are optimized with particle swarm optimization (PSO). Only the models in the best first 25% according to ROC AUC, Precision Recall AUC, Sensitivity, Specificity, MCC and F0.5 are kept. The model with the best F0.5 value is then chosen.

Supplemental Table 9: Different classification setups used for the classifier generation.

Feature scaling	Feature selection	Classifier
0-1 scaling	Select best 20 scoring features according to ANOVA F-value	Linear SVC
Mean 0, unit variance	Select best 50 scoring features according to ANOVA F-value	RBF SVC
None	Select features based on a linear SVC	Decision Tree
	Select features based on extremely randomized trees	Random Forest
	None	Extremely Randomized Trees
		Gradient Boosted Trees
		Extreme Gradient Boosted Trees
		AdaBoost
		Naive Bayes
		Linear Discriminant Analysis
		Quadratic Discriminant Analysis
		Nearest Neighbors
